# Using a fine-tuned large language model for symptom-based depression evaluation

**DOI:** 10.1038/s41746-025-01982-8

**Published:** 2025-10-07

**Authors:** Samantha Weber, Nicolas Deperrois, Robert Heun, Laura Frühschütz, Anna Monn, Stephanie Homan, Andrea Häfliger, Erich Seifritz, Tobias Kowatsch, MULTICAST consortium, Samantha Weber, Anna Monn, Stephanie Homan, Andrea Häfliger, Lena Jäger, Katharina Schultebraucks, Sapir Gershov, Jacopo Mocellin, Birgit Kleim, Sebastian Olbrich, Birgit Kleim, Sebastian Olbrich

**Affiliations:** 1 Psychiatric University Hospital Zurich, Department of Adult Psychiatry and Psychotherapy, Psychiatric University Clinic Zurich, Zürich, Switzerland; 2 Faculty of Medicine, University of Zurich, Zürich, Switzerland; 3 Department of Quantitative Biomedicine, University of Zürich, Zürich, Switzerland; 4 Department of Psychology, Experimental Psychopathology and Psychotherapy, University of Zurich, Zürich, Switzerland; 5 Institute for Implementation Science in Health Care, University of Zurich, Zurich, Switzerland; 6 School of Medicine, University of St. Gallen, St. Gallen, Switzerland; 7 Centre for Digital Health Interventions, Department of Management, Technology, and Economics, ETH Zurich, Zurich, Switzerland; 8 Department of Computational Linguistics, University of Zurich, Zurich, Switzerland; 9 Department of Psychiatry, New York University School of Medicine, New York, NY USA

**Keywords:** Diagnosis, Diagnostic markers

## Abstract

Recent advances in artificial intelligence, particularly large language models (LLMs), show promise for mental health applications, including the automated detection of depressive symptoms from natural language. We fine-tuned a German BERT-based LLM to predict individual Montgomery-Åsberg Depression Rating Scale (MADRS) scores using a regression approach across different symptom items (0–6 severity scale), based on structured clinical interviews with transdiagnostic patients as well as synthetically generated interviews. The fine-tuned model achieved a mean absolute error of 0.7–1.0 across items, with accuracies ranging from 79 to 88%, closely matching clinician ratings. Fine-tuning resulted in a 75% reduction in prediction errors relative to the untrained model. These findings demonstrate the potential of lightweight LLMs to accurately assess depressive symptom severity, offering a scalable tool for clinical decision-making, and monitoring treatment progress, particularly in low-resource settings.

## Introduction

Major depressive disorder is a leading global health concern^1^. Recent research has explored language-based behavioral markers to assess depressive symptoms focusing on prosodic, lexical, and (morpho)syntactic features^2^, as well as written language in contexts of medical notes or social media posts^3,4^. In parallel, artificial intelligence (AI) advancements have contributed significantly to the field, particularly in improving the performance of predictive models of depressive symptom-related factors, even though its superiority over other methods has not yet been proven^5,6^. While conventional AI techniques have proven valuable for neuroscience and psychiatric research, they might still struggle with long-range dependencies of language-based data - or in other words: the semantic context. The recent development of large language models (LLMs) - with their ability to extract information from natural language and to generate human-like texts - revolutionized the AI field and quickly adapted to diverse medical domains^7^, including mental health^8–10^. While LLMs excelled at tasks like depression detection^10^ or automatizing discharge summaries^8^, current research indicated that they cannot - at this stage - fully replicate specialized clinical reasoning as required, for example, in depressive symptom assessments.

However, despite these new advances in natural language processing (NLP) and AI, one of the greatest challenges in detecting and understanding clinical symptoms lies in capturing and accurately interpreting the nuanced, subjective nature of depressive symptoms expressed in natural language^11^. In the clinical daily routine, one way of quantifying depressive symptomatology is the Montgomery-Åsberg Depression Rating Scale (MADRS^12^), a structured clinical interview in which the clinician asks about ten different items representing core symptoms of depression. The patient’s narratives based on free speech make the MADRS clinical interview a perfect candidate for LLM-based automatization. In this study, we aimed to train and evaluate a German BERT (Bidirectional Encoder Representations from Transformers)-based model for predicting MADRS scores using a regression approach that captures the continuous nature of symptom severity. We compared the performance of the fine-tuned model with the base model to accurately predict MADRS subscores, highlighting that pre-trained models often exhibit unspecific predictions when applied to specialized data. In contrast, fine-tuning allows the model to adapt to a specific context, such as clinical data, which might be underrepresented or absent in the training datasets of general LLMs. This study contributes to the clinical and research applications of LLMs, particularly for the automated assessment and monitoring of depressive symptomatology in everyday life.

## Results

### Data acquisition and preprocessing

**Fig. 1:**
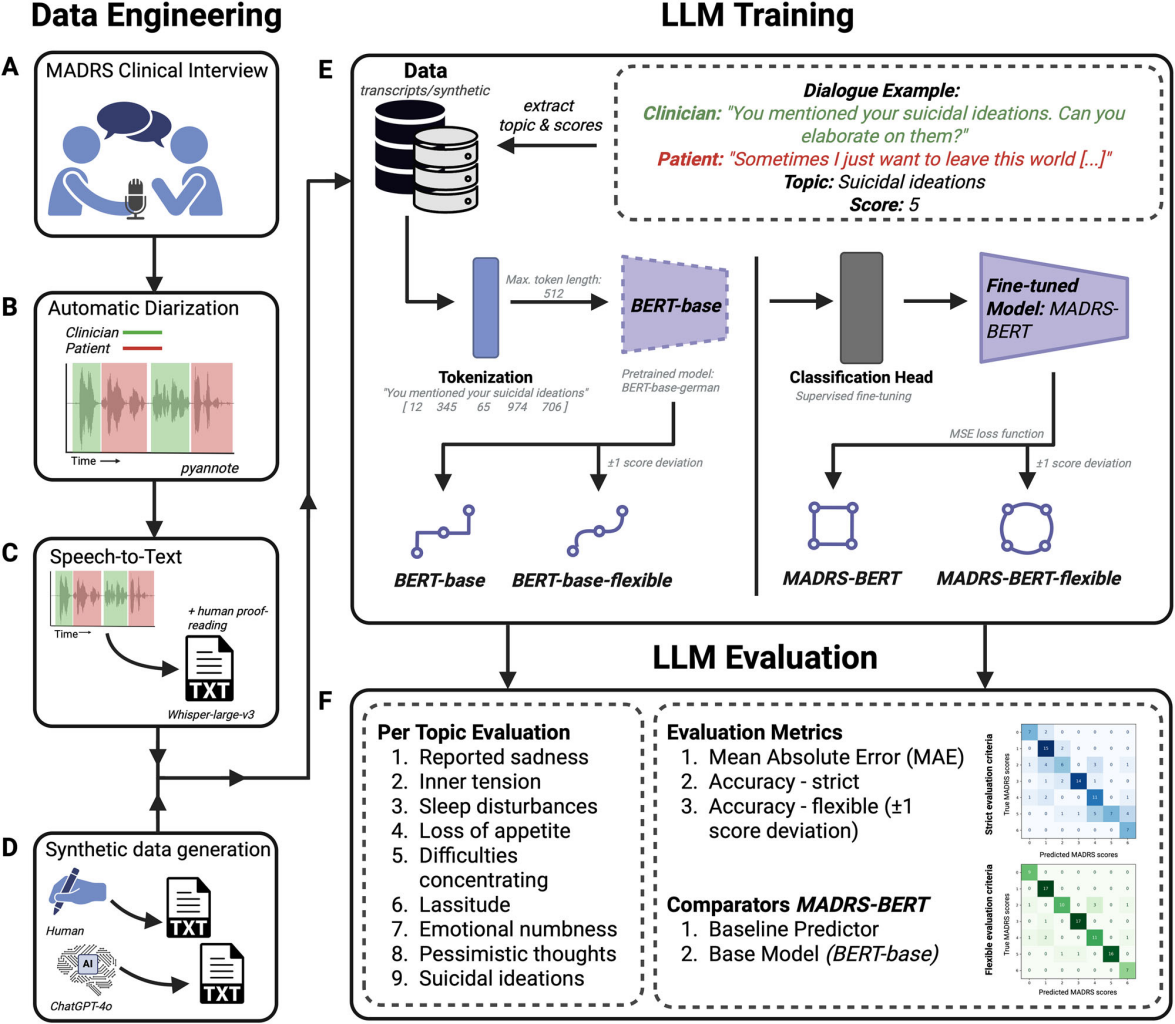
Workflow. Data Engineering. **A** MADRS clinical interviews were videotaped, and audio files were extracted. **B** Automatic speaker diarization was conducted using *pyannote* to segment audio files into hypothesized sequences of the individual speakers. **C** Individual segments were then transcribed using *Whisper-large-v3*. Transcripts were proofread, and items and scores were manually assigned. **D** To account for the unbalanced score distribution, further interviews were generated, including the underrepresented scores across the nine items. LLM Training: **E** Real patient transcript data and synthetic data were merged, tokenized, and used for training the pre-trained BERT-base-german model. A *flexible* evaluation metric was included to account for predictions within the ± 1 score deviation of the true label reflecting the practical application of the MADRS scoring in the clinical setting. LLM Evaluation: **F** The model was evaluated for each item individually using accuracy and mean absolute error (MAE), and confusion matrices were generated to illustrate *strict* and *flexible* predictions. Created in https://BioRender.com.

**Fig. 2:**
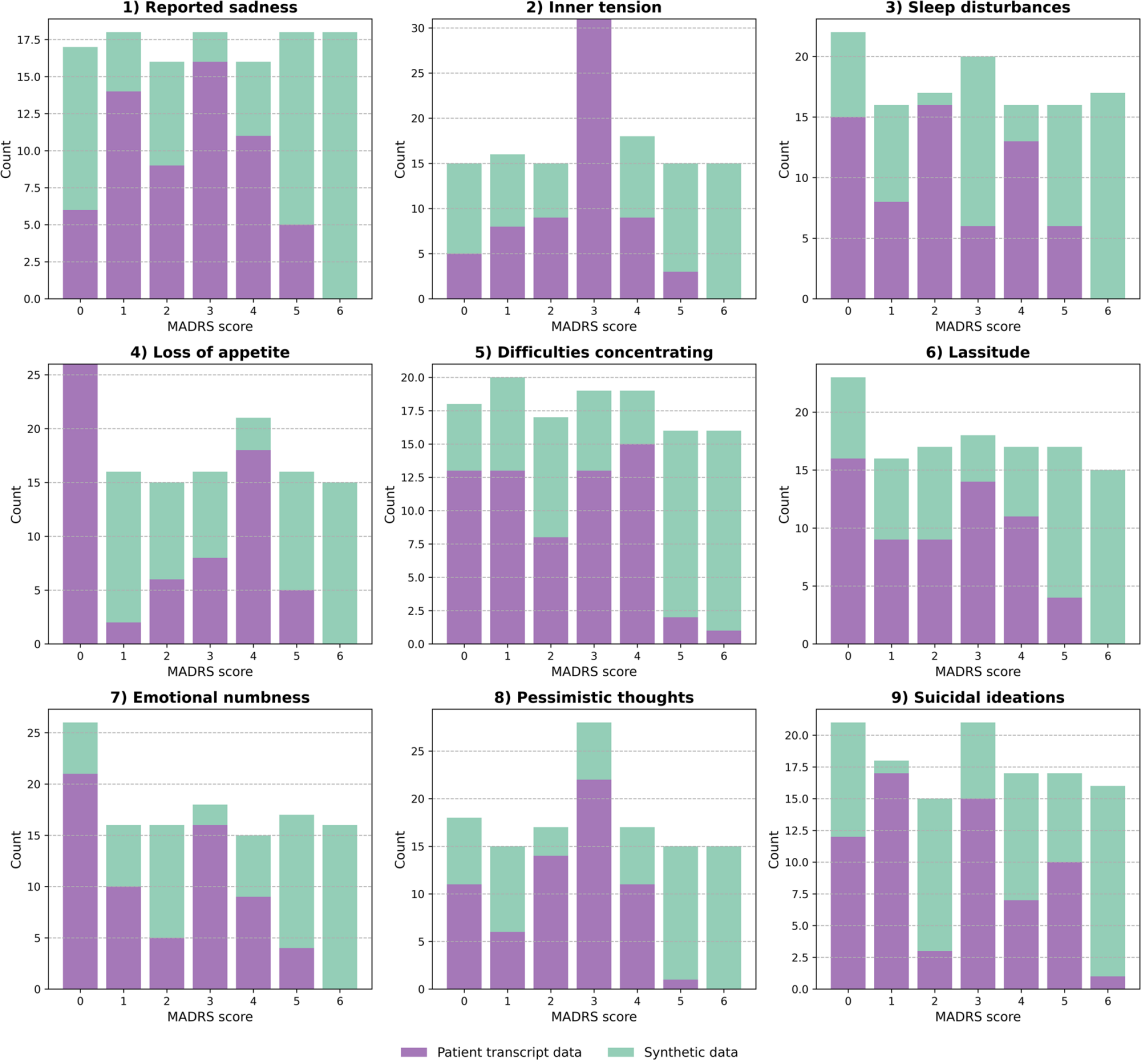
Distribution of MADRS scores across patient transcript data and synthetic data. The first item (“apparent sadness”) was excluded from the analysis as its rating depends not only on language but also on body language, posture, and facial mimics. Patient transcript data are shown in purple, and synthetic data is shown in light teal. Barplots represent counts of unique scores per item and subject. Abbreviations: MADRS: Montgomery-Åsberg Depression Rating Scale.

For fine-tuning and evaluation of our *MADRS-BERT* model, we prepared a structured, item-level dataset derived from transcribed and segmented patient interviews, focusing on nine core depressive symptom domains assessed by the MADRS while excluding “Apparent Sadness”, which requires non-verbal cues. To ensure a balanced score distribution, we combined real patient interviews with synthetically generated interviews. An overview of the data collection, preprocessing, and model fine-tuning pipeline is provided in Fig. 1. The dataset consisted of a total of 126 MADRS interviews (65 patient transcripts, 61 synthetic interviews; see Supplement for demographics). The 126 interviews resulted in 1’242 item-level samples for training and validation. The distribution of scores across real and synthetic data is presented in Fig. 2.

### Model performance

To evaluate model performance in predicting depressive symptom severity, we applied a regression-based fine-tuning approach on item-level data using a fivefold cross-validation approach. We assessed performance using mean absolute error (MAE) as a measure of prediction precision and accuracy, the latter computed by rounding continuous predictions to the nearest integer for comparison to clinician-assigned scores. Additionally, we applied a flexible evaluation criterion that considers predictions within ±1 point of the true label as correct, reflecting clinical tolerance for minor rating discrepancies.

First, in Table 1, we compared the regression performance of the fine-tuned model (*MADRS-BERT*) to the mean regression model (baseline prediction). Fine-tuning the model led to an MAE ranging from 0.7 for the item “inner tension” to 1.0 for the item “emotional numbness” across the nine items. Compared to the mean regression model (baseline predictor), fine-tuning reduced the MAE in average by 0.9 points. Second, we compared the performance of the fine-tuned model (*MADRS-BERT*) to the base model (BERT-base-German-cased; *“BERT-base”*) with no further task-specific fine-tuning. Accuracies under flexible evaluation criteria ranged from 79% to 88% across the nine items for *MADRS-BERT*. As expected, the base model exclusively predicted a score of 0 across all items and scores (Supplementary Fig. 4–5), indicating a complete lack of specificity for the task, that is, inability to differentiate between different levels of symptom severity.

**Table 1:** Comparison of *MADRS-BERT* and Baseline Predictor (Mean Regression Model) Performance Across MADRS items

MADRS Item	Mean MADRS Score	MAE ↓ (±std)	
		Baseline predictor	*MADRS-BERT*
Reported sadness	3.0	1.7	0.9 (±0.04)
Inner tension	3.0	**1.5**	**0.7** (±0.15)
Sleep disturbances	2.9	1.7	0.9 (±0.16)
Loss of appetite	2.8	1.8	0.8 (±0.08)
Difficulties concentrating	2.9	1.7	0.8 (±0.22)
Lassitude	2.8	1.8	0.8 (±0.21)
Emotional numbness	2.8	1.8	1.0 (±0.26)
Pessimistic thoughts	2.9	1.6	0.8 (±0.14)
Suicidal ideations	2.9	1.7	0.8 (±0.16)
Total	2.89	1.70	0.83

The table reports the Mean Score, and Mean Absolute Error (MAE) for the baseline predictor and the fine-tuned model (*MADRS-BERT*) across all nine MADRS items. The baseline predictor assigns the mean MADRS score per topic as the predicted value, serving as a naive statistical reference. MAE quantifies the prediction error, with lower values indicating better performance. Bold numbers highlight the best results.

**Fig. 3:**
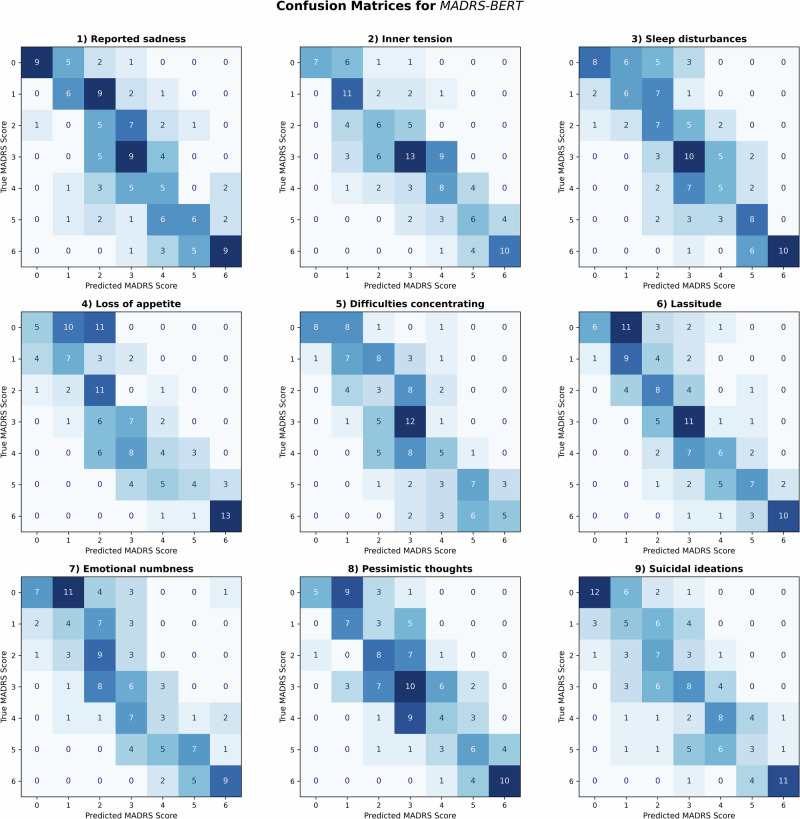
Confusion matrices for *MADRS-BERT* model. The confusion matrices illustrate the model performances and errors across the nine items using the fine-tuned *MADRS-BERT* model by comparing the predicted (x-axis) versus the actual (*y*-axis) MADRS scores. The intensity of the color represents the count of predictions, with darker shades indicating higher values. Diagonale entries represent correctly classified instances, while off-diagonal entries indicate errors.

**Fig. 4:**
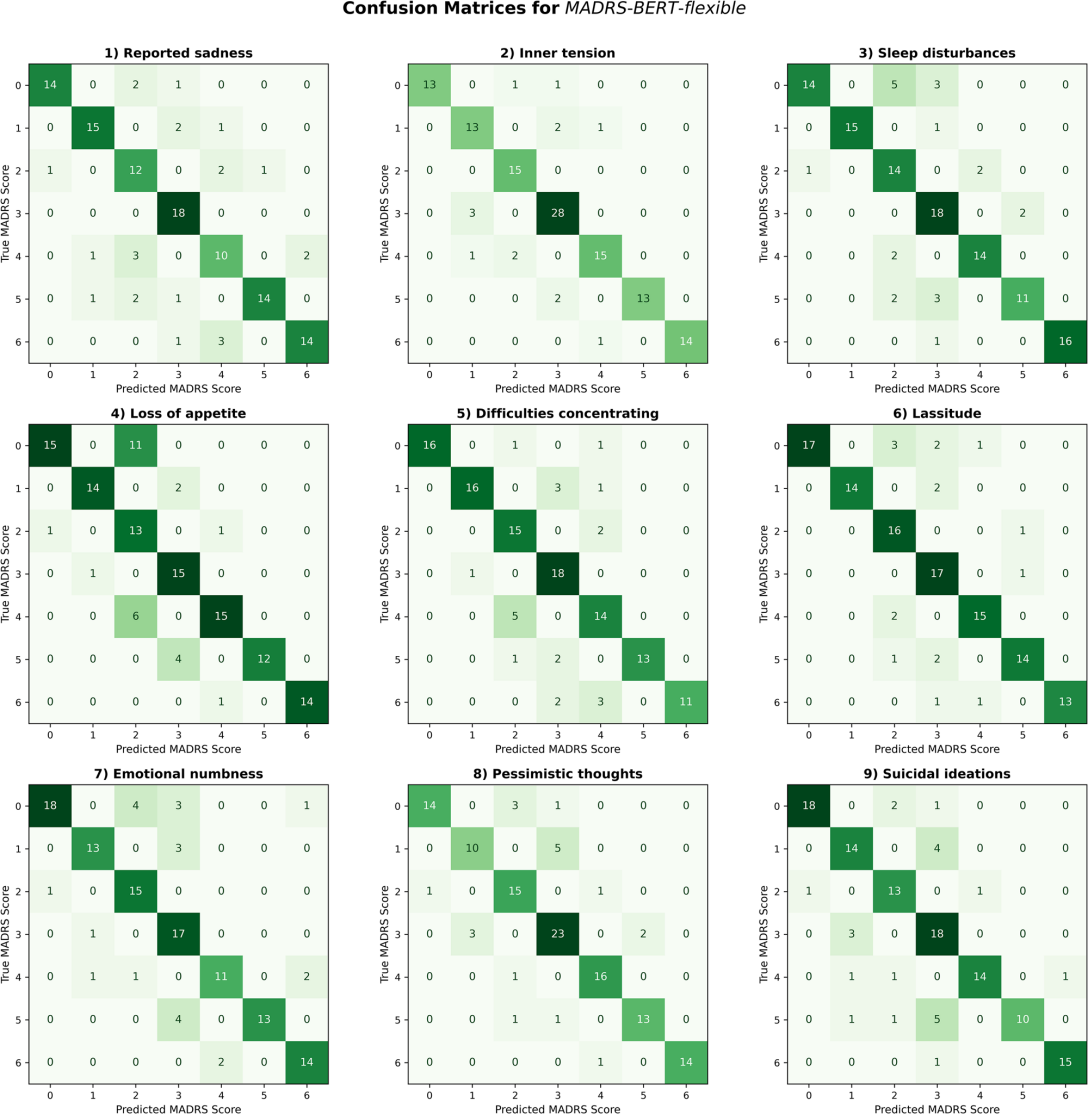
Confusion matrices for *MADRS-BERT-flexible* model. The confusion matrices illustrate the model performances and errors across the nine items using the fine-tuned *MADRS-BERT* model by comparing the predicted (*x*-axis) versus the true (y-axis) MADRS scores. The intensity of the color represents the count of predictions, with darker shades indicating higher values. Diagonale entries represent correctly classified scores, while off-diagonal entries indicate errors. The model’s performance is shown under the flexible criteria, with predictions within ±1 of the true label considered as a correct prediction.

Table 2 shows the accuracies across items for the fine-tuned (*MADRS-BERT*) and base (*BERT-base*) models under strict and flexible criteria. Figures 3 and 4 depict the corresponding confusion matrices for the fine-tuned model (*MADRS-BERT*) under strict and flexible criteria showing a clear distribution along the diagonal indicating the ability to differentiate between different symptom severity levels based on linguistic data (see Supplementary Figs. 4-5 for the confusion matrices of *BERT-base*). Each matrix reflects model predictions at the item level (that is, per text segment corresponding to a specific MADRS item) aggregated across all five validation sets from cross-validation. Overall, *MADRS-BERT* achieved high predictive accuracy across all nine items, significantly outperforming baseline models in capturing item-specific severity.

**Table 2:** Performance metrics of the fine-tuned *MADRS-BERT* and *BERT-base* models under strict and flexible criteria for accuracy

	*MADRS-BERT*		*BERT-base*	
MADRS Item	Accuracy ↑ [%] *Flexible*	Accuracy ↑ [%] *Strict*	Accuracy ↑ [%] *Flexible*	Accuracy ↑ [%] *Strict*
Reported sadness	80 ( ± 0.03)	40 ( ± 0.07)	29 ( ± 0.04)	14 ( ± 0.03)
Inner tension	**88 (** ± **0.06)**	**49 (** ± **0.10)**	25 ( ± 0.04)	12 ( ± 0.07)
Sleep disturbances	82 ( ± 0.08)	44 ( ± 0.09)	30 ( ± 0.07)	17 ( ± 0.07)
Loss of appetite	79 ( ± 0.04)	43 ( ± 0.12)	**33 (** ± **0.06)**	**20 (** ± **0.07)**
Difficulties concentrating	83 ( ± 0.08)	40 ( ± 0.14)	31 ( ± 0.06)	15 ( ± 0.06)
Lassitude	86 ( ± 0.07)	46 ( ± 0.16)	31 ( ± 0.09)	19 ( ± 0.08)
Emotional numbness	80 ( ± 0.12)	35 ( ± 0.11)	33 ( ± 0.11)	20 ( ± 0.08)
Pessimistic thoughts	85 ( ± 0.07)	41 ( ± 0.10)	26 ( ± 0.04)	14 ( ± 0.05)
Suicidal ideations	83 ( ± 0.10)	44 ( ± 0.10)	32 ( ± 0.05)	17 ( ± 0.04)

Mean and standard deviation of accuracies across five folds. Strict evaluation for accuracy considers exact score predictions, while flexible evaluation allows a deviation of ±1 from the actual score. Bold numbers highlight the best results.

### Error Analysis

Overall, the fine-tuned models outperformed the base models significantly. By focusing on errors beyond the ±1 range from the diagonal (flexible model evaluation), fine-tuning the model resulted in an overall 75.38% reduction of errors compared to the base model, and 30.29% reduction for the strict evaluation criteria. Under flexible evaluation criteria, the fewest prediction errors were observed in the item “inner tension”. Conversely, the highest error rate occurred in the item “loss of appetite.” Under strict evaluation criteria, the fewest errors were observed in the item “inner tension” and the highest error rate in the item “emotional numbness”.

### Scaling Model Performance with Data Availability

**Fig. 5:**
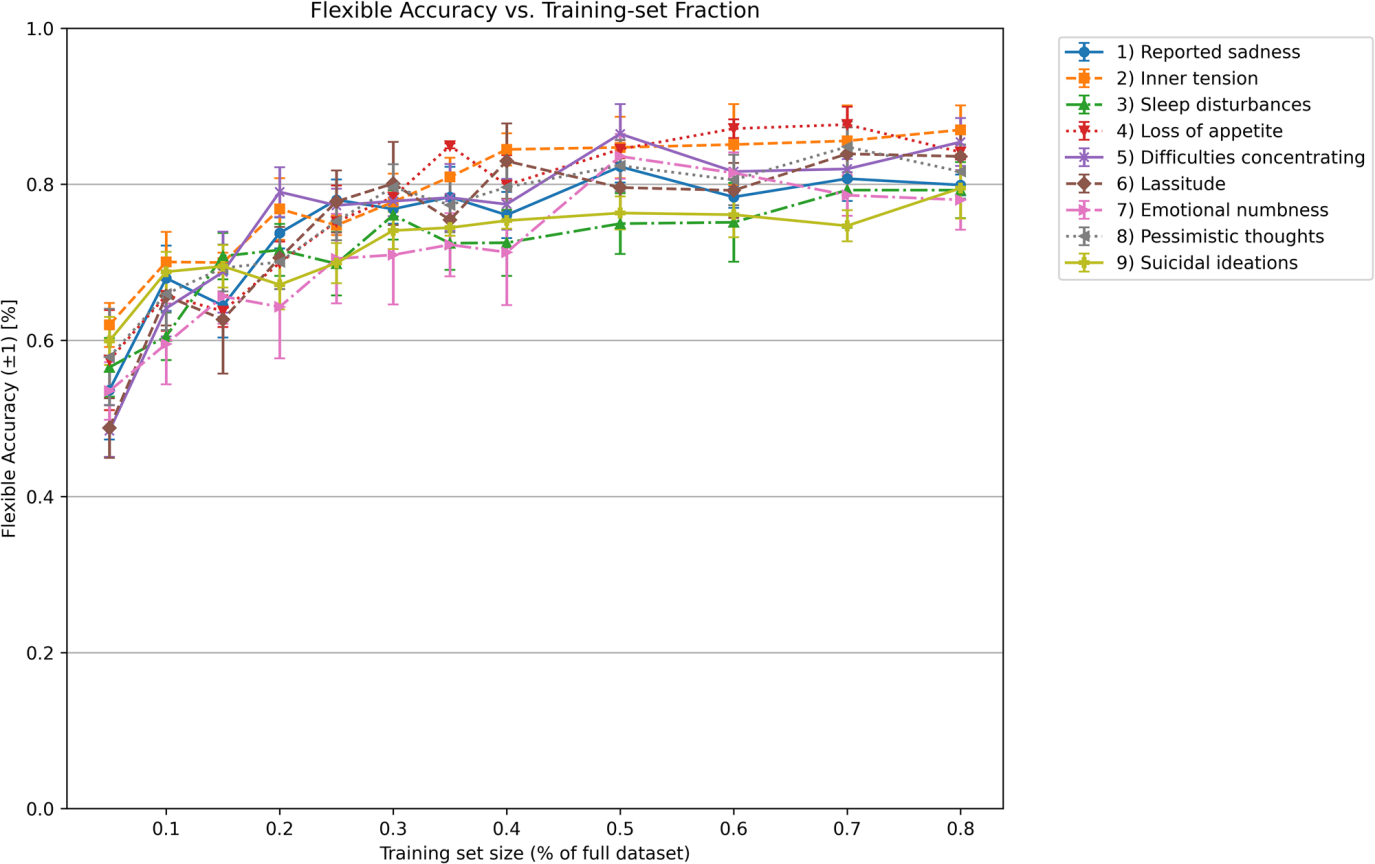
Learning curves for nine MADRS topics under the flexible accuracy criterion. Each line corresponds to one MADRS item. For each outer cross-validation fold, models were trained on increasing fractions (5–80%) of the entire dataset, with evaluation always performed on a fixed validation set comprising 20% of the full dataset. The *x*-axis indicates the proportion of the full dataset used for training, while the *y*-axis shows the mean flexible accuracy ( ± 1) across outer folds. Error bars indicate the standard error of the mean (SEM) across folds. Flexible accuracy considers predictions correct if they fall within ±1 of the true score, reflecting clinically acceptable variation.

To assess how performance scales with data availability, we performed a fivefold cross-validation across the full dataset. In each fold, we trained models on increasing fractions of the entire dataset (from 5 to 80%) and evaluated them on the fixed outer validation set (20%). As shown in Fig. 5, the learning curve for flexible accuracy showed a rapid performance increase up to approximately 50–80% of the data, after which improvements plateaued across most topics. This trend suggests that the model learns efficiently from the initial training data, but further gains may require either significantly more data or architectural adjustments.

## Discussion

In this study, we evaluated the prediction performance of a BERT-based LLM fine-tuned on German MADRS interview data and its corresponding scoring. Following fine-tuning, the model exhibited excellent performance with an average MAE of 0.7 to 1.0 and accuracies ranging from 79% to 88% across the nine items, resulting in a 75.38% error reduction. This demonstrates the fine-tuned model’s ability to match the clinicians’ ratings consistently. First, *MADRS-BERT* achieved an average reduction in MAE of 0.9 compared to the baseline predictor, highlighting its ability to generate precise and clinically meaningful severity estimates. Furthermore, as expected, when randomly initializing the linear output of BERT (*BERT-base*), the model is unable to correctly predict the labels showing a complete lack of specificity for the different levels of symptom severity. These findings demonstrate the substantial improvements resulting from fine-tuning, underscoring the model’s increased specificity, tailoring it to the requirements of the MADRS scoring, and substantially decreasing MAE and improving prediction accuracy.

Our approach stands in contrast to previous research that either predicted overall MADRS depression scores^13,14^ or employed broader binary classification mechanisms to detect general depression risk^15–17^. Notably, only a few studies have explored the use of LLMs in the context of structured clinical interviews using the MADRS scale. For instance, models evaluated on Italian MADRS interviews using prompt engineering achieved a maximum item-level accuracy of 30% to 60%, struggling with consistency across individual symptoms^13^. Similarly, in a Japanese dataset, LLMs trained on a large corpus of MADRS dialogs demonstrated strong correlation with total clinician-assigned scores (*r* = 0.86), yet focused exclusively on overall severity rather than the more granular symptom-level predictions central to our approach^14^.

Other studies have largely relied on alternative depression assessments such as the patient health questionnaire (PHQ-9^18^) or general risk detection frameworks. For example, binary classification approaches applied to unstructured text or diary entries achieved high accuracy (~90%) when fine-tuned, but base models without adaptation often failed to detect depressive signals reliably^15,16^. While promising, these methods generally lack the clinical specificity and interpretability afforded by structured rating scales. Similarly, zero-shot approaches using LLMs to estimate depression severity from clinical notes or patient narratives - such as mapping free-text input to PHQ-9 scores - have shown only moderate agreement with human ratings and inconsistent item-wise performance^19,20^. In contrast, *MADRS-BERT* was explicitly fine-tuned to predict continuous scores for each MADRS item based on expert clinical-ratings, enabling a structured assessment of depressive symptoms. This allows for the detection of nuanced changes in mood and behavior across specific domains such as pessimistic thoughts, inner tension and suicidal ideation - features that might be overlooked in broad binary frameworks. By aligning predictions with the MADRS structure, our model mirrors psychiatric practice and offers more clinically interpretable outputs. This item-level-based approach addresses key gaps in existing work by providing fine-grained, symptom-specific insights that could support both research applications and real-world clinical symptom assessment and monitoring. Moreover, it enhances the transparency and utility of LLM-based systems by mapping outputs onto a validated psychiatric instrument - a feature highlighted as essential for clinical trust and decision making in recent reviews^21,22^.

Beyond these improvements in predictive performance, our study also addresses broader methodological and implementation challenges discussed across recent literature, particularly regarding generalizability, explainability, and clinical scalability^21,23^. Generalizability remains a critical issue in mental health AI, especially when models are trained on small or imbalanced datasets. To avoid overfitting, we employed a *K*-fold cross-validation, a strategy endorsed for psychiatric NLP applications where large held-out test datasets are rarely available^23^. Moreover, *MADRS-BERT* was designed to generate a more fine-grained prediction of symptom severity estimates, moving beyond the limitations of traditional binary classification models that often oversimplify depression into a present-absent dichotomy. In line with recent efforts by Lau et al.^24^ who modeled depression severity as a continuous regression task using parameter-efficient tuning, we similarly adopted a continuous scoring framework based on a regression model and evaluated the model performance using MAE, a clinically interpretable metric. Both approaches highlight the clinical relevance of moving beyond binary classification toward more nuanced, symptom-level assessments. Additionally, Lau et al. conducted a learning curve analysis, which - like ours - demonstrated substantial performance gains up to 80% of the training data, followed by a plateau^24^. Similarly, recent work by Huang et al. using a regression framework on self-supervised voice-based pretraining models illustrated that lightweight, modality-specific models can offer strong performance for depression assessment^25^. This convergence across independent studies suggests that even lightweight models, when fine-tuned with high-quality clinical data, can achieve robust performance and that additional performance gains may depend on further modality integration or more complex model architectures. In line with our efforts, Lorge et al. showed that training an LLM with synthetic clinical text can effectively bypass data scarcity and privacy limitations^26^ - a strategy we also adopted by generating interviews to supplement underrepresented MADRS classes. Their BERT-based span extraction model for difficult-to-treat depression, trained on synthetic data, provided strong evidence that synthetic data augmentation is a scalable solution for LLM training in mental health care settings^26^. Overall, these findings underscore the growing consensus around building interpretable, generalizable, and deployment-ready AI tools optimized for real-world use^21,23,27^.

Our findings also speak to ongoing calls for more transparent and explainable AI in psychiatry^28^. As highlighted in recent reviews^22^, clinicians and researchers must be able to interpret and trust model outputs. Our work supports the scalability of structured, language-based assessments by generating clinically aligned outputs that are both interpretable and reproducible. By integrating the structured nature of the MADRS interview, *MADRS-BERT* generates interpretable and standardized outputs that can be used consistently over time for longitudinal tracking - thus supporting both clinical workflows and research designs. Consistent with prior discussions^23^, such systematic tools can reduce stigma, cost, and time barriers in mental health services, while improving diagnostic assessments in remote or low-resource mental health care settings.

Several promising directions may further improve model robustness and broaden real-world applicability. Recent advances in multimodal LLMs have already demonstrated the feasibility of combining linguistic and facial features for depression detection^29^, and previous work has highlighted the role of prosodic, spectral, and voice-quality cues in improving model performance^23^. Their clinical validation and integration into real-world psychiatric workflow remain an important next step^30^. Particularly in the care of depression, LLMs become increasingly useful not only for screening and diagnosing, but also for patient management and monitor depressive symptomatology over time^21,23,31,32^. Beyond structured symptom tracking, several emerging strategies could further enrich LLM-based assessments like *MADRS-BERT*. For instance, retrieval-augmented generation has recently been used to support psychiatric decision-making by grounding model outputs in contextualized patient data from electronic health records^33^. While such systems are still in early stages, they illustrate how augmenting LLMs with individualized context could enhance clinical relevance without sacrificing interpretability. Likewise, incorporating free-text rationales alongside item-level predictions has been proposed as a way to increase transparency and model robustness, offering clinicians insight into how scores are derived^34^. While not prerequisites for clinical deployment, these strategies may complement symptom-focused models like *MADRS-BERT* in the future, especially as explainability becomes a growing priority in clinical AI.

In summary, integrated into digital screening or patient monitoring technologies^35^, LLMs offer new, scalable directions for assessing symptoms in a standardized manner in research studies and in the clinical routine. They may provide diagnostic support, assist in symptom monitoring over time, and ultimately contribute to reducing treatment costs while improving accessibility^30,35^. However, as recent studies highlighted^21,36^, LLMs in mental health care must be deployed with caution, particularly in high-risk scenarios. Structured tools like *MADRS-BERT*, which focus on fine-grained, clinician-aligned assessments within defined rating instruments, may offer a more controlled and interpretable starting point for real-world implementation than general-purpose models. Building on this foundation, future research should explore hybrid systems that balance automation with human oversight, while continuing to evaluate explainability and safety in diverse clinical populations.

Our study has several limitations. We applied a novel approach by integrating flexibility into the model’s prediction, a feature that aligns closely with clinical practice, where slight discrepancies between raters are common due to human interpretation and variability^37^. Bridging the gap between strict data-driven analyses and real-world clinical practice, allowing the model a ± 1 deviation enhances the model’s clinical applicability and aligns with previously reported practical flexibility, where intra-class correlation values ranged between 0.866 and 0.978 for each item of the MADRS^37^. However, while this brings practical benefits, it also introduces the risk of clinically significant misclassification, which we must account for when interpreting model performance. Further limitations may include our handling of discrepancies between the two raters. Additional limitations concern how rater disagreements were handled. When disagreements exceeded ±1 on individual ratings or four points on the total score occurred, a consensus rating was applied. Since the model was evaluated on the individual items, the consensus procedure for broader disagreements between raters could not be incorporated. Furthermore, we could not report formal inter-rater reliability metrics as individual rater scores before the consensus procedure were not consistently stored for retrospective analyses. However, we assume that the consensus rating reflects shared clinical judgment and is thus appropriate as ground truth for model training and evaluation. Although the sample size seemed adequate, a larger dataset may increase the generalizability of the model. We addressed this by generating additional synthetic data to balance the uneven class distributions. However, this approach also reduces the baseline difficulty, as a mean-regression model can achieve reasonable performance by predicting the average score, limiting its clinical relevance when the data distribution is balanced across all severity levels. An important limitation to our approach is that repeat interviews from the same participant were treated as independent samples and not grouped during fold assignment. As a result, data points from the same individual may appear in both training and validation folds, which could introduce a small degree of dependence. However, as these follow-up interviews were conducted weeks apart and reflect distinct clinical states, we considered them to provide non-identical information relevant to the generalization of the model. Our design choice allowed for more efficient use of the available dataset and reflects realistic clinical variability. Moreover, the MADRS interview format focuses on symptom description rather than personal narrative. Thus, repeated samples rarely contain personally identifying content, and item-level modeling further reduces the risk of the model memorizing individual language patterns. While our approach offers a light-weight solution, future work should explore the use of larger generative models, which offer the capabilities of in-context learning or few-shot adaptations that might eliminate the need for task-specific fine-tuning. Moreover, while proven powerful, LLMs lack the ability to focus on non-verbal cues (as required for item 1: “Apparent sadness”), and the integration of multimodal LLMs for clinical purposes must be explored^30^. Lastly, the pretraining data might introduce a bias potentially favoring “WEIRD” populations (westernized, educated, industrialized, rich, and democratic), which affects the model’s performance in more diverse or non-Western contexts^38^. Along that line, we did not include any details on gender, age, sociodemographic heterogeneity, or cultural or hereditary differences in our analyses.

In conclusion, our study demonstrates the potential of LLM-based tools in accurately predicting MADRS scores for the individual items, offering a novel approach to assess depressive core symptoms in clinical practice and research suitable for deployment in low-resource environments such as healthcare settings. The high accuracy in predicting individual subscores might allow the assessment of depressive core symptoms using digital health technologies^35^, which could substantially help monitor treatment response or implement preventative measures. However, integrating state-of-the-art AI technologies into the clinical routine must be cautiously approached^21,36,39^. These technologies should aim to complement and not replace mental health care providers, prioritizing ethical considerations such as data privacy and security, which are key to optimal patient handling and well-being^21,40^.

## Methods

### Data acquisition and preprocessing

A total of 65 interviews were conducted in German or Swiss German. Twenty-one patients were interviewed twice with a 4–5 week interval between the two sessions (as a result of a follow-up interview after hospital discharge), and 23 participants were interviewed once. All interviews were videotaped and explicitly covered the past seven days. The items of the MADRS covered 0: apparent sadness, 1: reported sadness; 2: inner tension, 3: sleep disturbances, 4: loss of appetite, 5: difficulties concentrating, 6: lassitude, 7: emotional numbness, 8: pessimistic thoughts, and 9: suicidal ideations. The interviews followed the structure of the MADRS clinical interview^12^. For each item, the interviewer used a standardized probe question (e.g., “How has your sleep been this past week?”), and the patient was encouraged to respond freely in narrative form. Follow-up questions were asked to address the severity, duration or persistence of the symptoms, focusing on symptom description rather than personal narratives. Ratings were then assigned by the interviewers based on the open-ended responses (rating from 0 = ‘no symptoms’ to 6 = ‘most severe symptoms’ per item). The video-taped MADRS interviews were conducted by trained clinical researchers, including a certified psychotherapist (SH), a senior psychologist (AM), and trained psychology master’s students. The rating of those videos was done by the lead interviewer and second clinical researcher. Ratings were assigned independently and discussed immediately after the interview. If a disagreement of more than one point on the individual ratings or more than four points on the total score occurred, a consensus rating was decided upon. These consensus ratings were used for model training and evaluation. For further analysis, the first item (“0: apparent sadness”) was excluded as the scoring thereof depended not only on language, but also on facial mimics, body language, posture, and the patient’s general appearance.

### Audio files and automatic speech recognition

The audio was extracted from the video recordings using Python with *moviepy* libraries. To ensure consistency across recordings, the audio was subsequently processed using *pydub* to standardize to a single channel, reducing variability and ensuring compatibility with subsequent analysis workflow. We then performed speaker diarization to segment the audio into hypothesized sequences of the individual speakers within each interview using a pre-trained pipeline from *pyannote.audio* version 3.1(https://huggingface.co/pyannote/speaker-diarization-3.1) with default parameter settings. Segmenting the audio file based on speaker turns allowed the identification of individual speakers across the recordings. Then, each diarized segment was transcribed subsequently using *Whisper-large-v3* (OpenAI; https://huggingface.co/openai/whisper-large-v3), which has been proven to be a viable speech recognition system for the Swiss German language^41^. This method allowed for the automated reconstruction of the interviews in a structured dialog format, distinguishing between *clinician* and *patient* contributions. Filler and non-lexical words occurring naturally in spontaneous speech (e.g., “mmh”) were filtered out from the transcriptions. Given the challenges posed by Swiss German dialect variations and technical limitations of the diarization and automatic speech recognition, each transcription was then manually reviewed and corrected where necessary. Following transcription, interviews were manually segmented by item, based on the structure of the MADRS interview. For each item, the corresponding segment - comprising the relevant question and the patient’s response - was isolated and annotated. This manual segmentation ensured that each data sample used in later modeling corresponded precisely to one MADRS item. Lastly, each item-specific segment was then paired with its corresponding numeric score (0–6), based on the prior clinical rating by the trained interviewers.

### Generation of synthetic interviews

Synthetic interviews were generated to balance the score distribution by simulating interviews that included underrepresented scores across the nine items. This approach aims at improving the robustness and generalizability of the model evaluation (see *Model fine-tuning and performance evaluation*). The synthetic interviews were generated using a combination of manual creation by researchers and prompting of ChatGPT-4o to produce clinical interview texts. Interview texts where ChatGPT-4o was used, were further adapted and refined by researchers to ensure context sensitivity where needed. Overall, a total number of 61 synthetic interview transcripts were generated, which, together with the 65 interviews derived from real patient transcripts, allowed for a minimum of 15 interviews per score and item. To evaluate the similarity between real and synthetic data, we used cosine similarity (Supplementary Material).

### Model fine-tuning and performance evaluation

For MADRS score prediction, we fine-tuned a BERT-base-German-cased (https://huggingface.co/google-bert/bert-base-german-cased) model using a regression approach. This approach was selected based on the following rationale: First, although the MADRS produces discrete ratings, these values reflect a continuous spectrum of depressive symptom severity - capturing subtle variations in mood and behavior that can be clinically meaningful. Modeling symptom severity as a continuum using a regression offers a more nuanced approach than treating the scores as strictly categorical. Second, BERT-base-German-cased^42^ is a state-of-the-art, open-source model known for its strong performance in NLP classification tasks. We selected this model due to its effectiveness in capturing context, its availability in a pre-trained version suitable for our language-specific tasks and its relatively lightweight architecture (110 million parameters). Each data point in our model corresponds to a single MADRS item from an interview, treated independently of the full interview structure. We modeled each item separately to reflect item-level severity, resulting in 1’242 samples. As such, we did not use the full interview as input; rather, each model input was a single item-specific segment, paired with the previously assigned item-specific score. This item-level structure enabled symptom-specific prediction and aligned with the original interview format (an example of the data set structure can be found along with the published code and the model weights; see Data Availability Statement). To reflect the item-specific nature of the task, we retained the shared BERT encoder across all samples but added nine separate linear regression heads on top of the <CLS> token, that is, one for each MADRS item. This architecture enabled the model to specialize in scoring each symptom dimension while sharing contextual language knowledge across items. Notably, 21 participants contributed to two interviews, conducted as a follow-up interview 4–5 weeks after hospital discharge. These repeated interviews were treated as independent samples, reflecting potentially different symptom states.

The model was fine-tuned to predict MADRS subscores as a continuous variable (0–6) using a mean squared error (MSE) loss function, which penalizes larger deviation from true scores more strongly. Each regression head linearly projected the <CLS> token embedding to a single output neuron representing the predicted score. A BERT tokenizer was applied to the dialog-based input data, truncating and padding sequences to a maximum length of 512 tokens. We adapted a fivefold cross-validation approach using stratified *K*-fold splitting with random shuffling to ensure robust generalizability across items. Thus, each fold used approximately 80% of the data for training (~993 samples) and 20% for testing (~248 samples). Sessions were not grouped by participant IDs during fold assignment - meaning that samples from the same individual could appear in both training and test folds. Both real and synthetic interviews were included in training and evaluation. While these design choices increase the available sample size per fold and allows for symptom-specific evaluation, we acknowledge it may introduce a degree of non-independence and address this in the limitations. For each fold, a new instance of the BERT model was reinitialized. The model was fully fine-tuned (that is, no parameter-efficient approaches such as LoRA were used) using the AdamW optimizer with the following parameters: Learning rate=2e^-5^, weight decay = 0.01, batch size = 4, epochs = 15, gradient clipping = 0.5, warmup steps = 500, learning rate schedular = linear decay, early stopping = patience of five epochs (evaluated on validation loss). Training and evaluation were conducted locally on two NVIDIA GeForce 4090 GPUs, each with 24 GB VRAM.

We evaluated the model performance using the MAE (MAE; average absolute deviation between predicted and true score) and accuracy across all cross-validation folds (averaged) as an intuitive measure for overall performance. As the output of the regression model provides continuous data, accuracy was computed by first rounding it to the nearest integer. In addition to the standard evaluation metrics across the seven severity labels (0–6), we included a *flexible* evaluation criteria accounting for predictions within the ± 1 score deviation of the true label as acceptable. This approach serves two key purposes: First, MADRS scoring remains a subjective and noisy process, even among trained clinicians, so small deviations are often considered acceptable and described as commonly occurring in clinical practice^37^. Second, although the regression objective is designed to approximate the true score as closely as possible, a strict exact-score evaluation may not fully capture the practical utility of near-correct predictions in real-world clinical practice. Performance metrics were computed at the item level (that is, per sample) and averaged across all folds. Confusion matrices were constructed by aggregating item-level predictions across all five validation sets from cross-validation, separately for each MADRS topic, to visually represent the item-level performances and errors (Figs. 3–4). To assess the impact of fine-tuning, we compared the performance of our fine-tuned model (*MADRS-BERT*) against two benchmarks: First, to contextualize the performance of our fine-tuned model, we compared it against a naive baseline predictor (mean regression model) that assigns the mean MADRS score per topic as the predicted value and further calculate the MAE based on the true label and the artifically selected predicted value. While this approach does not involve any language understanding, it serves as a lower bound for performance - indicating the expected error if a model would simply rely on statistical tendencies based on the distribution of the data rather than meaningful linguistic patterns. Second, we compared it to the pre-trained base model (BERT-base-German-cased) without task-specific fine-tuning (that is, zero-shot evaluation). Doing so, we can assess whether fine-tuning successfully adapted the model to the clinical domain.

### Error analysis

We conducted an error analysis by comparing the number of incorrect predictions made by the base and fine-tuned models. The percentage error reduction was calculated by comparing the total errors of the base model to the total errors of the fine-tuned model to quantify the percentage decrease in misclassification achieved by the fine-tuned model.

$${Error\; Reduction}\left( \% \right)=\left(\frac{TotalError{s}_{BERT-base-flexible}-TotalError{s}_{MADRS-BERT-flexible}}{TotalError{s}_{BERT-base-flexible}}\right)\times 100$$

The methodological workflow is illustrated in Fig. 1.

### Scaling model performance with data availability

We examined how model performance scaled with training data size. For this, we conducted a fivefold cross-validation on the full dataset. In each outer fold, 80% of the data served as training pool and 20% as validation set. We created progressively larger subsets ranging from 5 to 80% of the entire dataset, ensuring that smaller fractions were strict prefixes of the larger ones to maintain consistency in sampling. Each subset was used to fine-tune a separate model, which was evaluated on the same fixed validation set of the corresponding fold. The resulting learning curve (Fig. 5) illustrates how performance scaled with data availability. The incremental performance for the strict evaluation criteria as well as the training and evaluation loss figures can be found in the Supplementary Figs. 1–3. Lastly, we performed the full experiment using real data only to assess the model’s generalizability to authentic clinical material only, independent of synthetic data augmentation (Supplementary Figs. 10 and 11, Supplementary Table 5).

### Declaration statements (1) Data availability

Real data derived from patient interview transcripts cannot be shared to ensure patient privacy. The synthetically generated data are available under: https://github.com/webersamantha/MADRS-BERT/data.

### Ethical considerations and participants

The study was carried out at the Psychiatric University Hospital Zurich, Switzerland, within the framework of a more extensive study on the identification of predictive markers for suicidal thoughts and behavior in a transdiagnostic cohort following discharge from inpatient psychiatric care (https://multicast.uzh.ch/en.html). The study was approved by the Ethics Committee of the University of Zurich (22.9.19) and conducted according to the Declaration of Helsinki. All participants provided written informed consent. Until December 2024, 66 patients were enrolled in the study. Inclusion criteria were 1) age between 18 to 65 years, 2) current or past suicidal thoughts and/or behaviors, and 3) being fluent in German or Swiss-German. Exclusion criteria were 1) a cognitive impairment due to acute psychoses, an intellectual disability, or dementia, 2) self- or other-directed aggression or violence, 3) pregnancy or breastfeeding, and 4) undergoing electroconvulsive therapy.

## Supplementary information

SupplementaryMaterial_LLM_MADRS_Weber

## Data Availability

Real data derived from patient interview transcripts cannot be shared to ensure patient privacy. The synthetically generated data is available under: https://github.com/webersamantha/MADRS-BERT/data. The code used for data preprocessing and fine-tuning is available under: https://github.com/webersamantha/MADRS-BERT. The model weights can be downloaded under: https://huggingface.co/webesama/MADRS-BERT.

## Code availability

Real data derived from patient interview transcripts cannot be shared to ensure patient privacy. The synthetically generated data is available under: https://github.com/webersamantha/MADRS-BERT/data.

## References

[1] Riera-Serra, P. et al. Clinical predictors of suicidal ideation, suicide attempts and suicide death in depressive disorder: a systematic review and meta-analysis. *Eur. Arch. Psychiatry Clin. Neurosci.* **274**, 1543–1563 (2024). 38015265 10.1007/s00406-023-01716-5 PMC11422269

[2] Homan, S. et al. Linguistic features of suicidal thoughts and behaviors: a systematic review. *Clin. Psychol. Rev.* **95**, 102161 (2022). 35636131 10.1016/j.cpr.2022.102161

[3] De Choudhury, M., Kiciman, E., Dredze, M., Coppersmith, G. & Kumar, M. Discovering shifts to suicidal ideation from mental health content in social media. In *Proc. CHI Conference on Human Factors in Computing Systems* 2098–2110 (ACM, 2016). 10.1145/2858036.2858207 PMC5659860 29082385

[4] Meyerhoff, J. et al. Analyzing text message linguistic features: do people with depression communicate differently with their close and non-close contacts? *Behav. Res. Ther.* **166**, 104342 (2023). 37269650 10.1016/j.brat.2023.104342 PMC10330918

[5] Corke, M., Mullin, K., Angel-Scott, H., Xia, S. & Large, M. Meta-analysis of the strength of exploratory suicide prediction models; from clinicians to computers. *BJPsych. Open* **7**, e26 (2021). 33407984 10.1192/bjo.2020.162 PMC8058929

[6] Levis, B., Benedetti, A. & Thombs, B. D. Accuracy of Patient Health Questionnaire-9 (PHQ-9) for screening to detect major depression: individual participant data meta-analysis. *BMJ* **365**, 1476 (2019). 10.1136/bmj.l1476 PMC6454318 30967483

[7] Malgaroli, M., Hull, T. D., Zech, J. M. & Althoff, T. Natural language processing for mental health interventions: a systematic review and research framework. *Transl. Psychiatry* **13**, 309 (2023). 37798296 10.1038/s41398-023-02592-2 PMC10556019

[8] Schwieger, A. et al. Large language models can support generation of standardized discharge summaries – a retrospective study utilizing ChatGPT-4 and electronic health records. *Int. J. Med. Inf.* **192**, 105654 (2024). 10.1016/j.ijmedinf.2024.105654 39437512

[9] Van Heerden, A. C., Pozuelo, J. R. & Kohrt, B. A. Global mental health services and the impact of artificial intelligence–powered large language models. *JAMA Psychiatry* **80**, 662 (2023). 37195694 10.1001/jamapsychiatry.2023.1253

[10] Xu, X. et al. Mental-LLM: leveraging large language models for mental health prediction via online text data. *Proc. ACM Interact. Mob. Wearable Ubiquitous Technol.* **8**, 1–32 (2024). 10.1145/3643540 PMC11806945 39925940

[11] Trifu, R. N. et al. Linguistic markers for major depressive disorder: a cross-sectional study using an automated procedure. *Front. Psychol.* **15**, 1355734 (2024). 38510303 10.3389/fpsyg.2024.1355734 PMC10953917

[12] Montgomery, S. A. & Åsberg, M. A new depression scale designed to be sensitive to change. *Br. J. Psychiatry* **134**, 382–389 (1979). 444788 10.1192/bjp.134.4.382

[13] Raganato, A. et al. Leveraging prompt engineering and large language models for automating MADRS score computation for depression severity assessment. **4**, e0000943 (2024).

[14] Shimamoto, M. et al. Machine learning algorithm-based estimation model for the severity of depression assessed using Montgomery-Asberg depression rating scale. *Neuropsychopharmacol. Rep.* **44**, 115–120 (2024). 38115795 10.1002/npr2.12404 PMC10932776

[15] Lorenzoni, G., Velmovitsky, P. E., Alencar, P. & Cowan, D. GPT-4 on clinic depression assessment: an LLM-based pilot study. In *Proc.**IEEE International Conference on Big Data (BigData)* 5043–5049 (IEEE, 2024).

[16] Shin, D., Kim, H., Lee, S., Cho, Y. & Jung, W. Using Large language models to detect depression from user-generated diary text data as a novel approach in digital mental health screening: instrument validation study. *J. Med. Internet Res.* **26**, e54617 (2024). 39292502 10.2196/54617 PMC11447422

[17] Zhang, Y. et al. Identifying depression-related topics in smartphone-collected free-response speech recordings using an automatic speech recognition system and a deep learning topic model. *J. Affect. Disord.* **355**, 40–49 (2024). 38552911 10.1016/j.jad.2024.03.106

[18] Kroenke, K., Spitzer, R. L. & Williams, J. B. W. Patient Health Questionnaire-9. **16**, 606–613 (2011).

[19] McCoy, T. H., Castro, V. M. & Perlis, R. H. Estimating depression severity in narrative clinical notes using large language models. *J. Affect. Disord.* **381**, 270–274 (2025). 40187432 10.1016/j.jad.2025.04.014 PMC13489508

[20] Lho, S. K. et al. Large language models and text embeddings for detecting depression and suicide in patient narratives. *JAMA Netw. Open* **8**, e2511922 (2025). 40408109 10.1001/jamanetworkopen.2025.11922 PMC12102709

[21] Omar, M. & Levkovich, I. Exploring the efficacy and potential of large language models for depression: a systematic review. *J. Affect. Disord.* **371**, 234–244 (2025). 39581383 10.1016/j.jad.2024.11.052

[22] Teferra, B. G. et al. Screening for depression using natural language processing: literature review. *Interact. J. Med. Res.* **13**, e55067 (2024). 39496145 10.2196/55067 PMC11574504

[23] Mao, K., Wu, Y. & Chen, J. A systematic review on automated clinical depression diagnosis. *npj Ment. Health Res.* **2**, 20 (2023). 38609509 10.1038/s44184-023-00040-z PMC10955993

[24] Lau, C., Zhu, X. & Chan, W.-Y. Automatic depression severity assessment with deep learning using parameter-efficient tuning. *Front. Psychiatry* **14**, 1160291 (2023). 37398577 10.3389/fpsyt.2023.1160291 PMC10308283

[25] Huang, X. et al. Depression recognition using voice-based pre-training model. *Sci. Rep.* **14**, 12734 (2024). 38830969 10.1038/s41598-024-63556-0 PMC11637030

[26] Lorge, I. et al. Detecting the clinical features of difficult-to-treat depression using synthetic data from large language models. *Comput. Biol. Med.* **194**, 110246 (2025). 10.1016/j.compbiomed.2025.110246 40499374

[27] Lundberg, S. M. & Lee, S.-I. A unified approach to interpreting model predictions. **30**, 4768–4777 (2017).

[28] Joyce, D. W., Kormilitzin, A., Smith, K. A. & Cipriani, A. Explainable artificial intelligence for mental health through transparency and interpretability for understandability. *npj Digit. Med.* **6**, 6 (2023). 36653524 10.1038/s41746-023-00751-9 PMC9849399

[29] Sadeghi, M. et al. Harnessing multimodal approaches for depression detection using large language models and facial expressions. *npj Ment. Health Res.* **3**, 66 (2024). 39715786 10.1038/s44184-024-00112-8 PMC11666580

[30] Stade, E. C. et al. Large language models could change the future of behavioral healthcare: a proposal for responsible development and evaluation. *npj Ment. Health Res.* **3**, 12 (2024). 38609507 10.1038/s44184-024-00056-z PMC10987499

[31] Borentain, S. et al. Montgomery-Åsberg depression rating scale factors in treatment-resistant depression at onset of treatment: derivation, replication, and change over time during treatment with esketamine. *Int. J. Methods Psychiatr. Res.* **31**, e1927 (2022). 35749277 10.1002/mpr.1927 PMC9720209

[32] Goh, E. et al. Large language model influence on diagnostic reasoning: a randomized clinical trial. *JAMA Netw. Open* **7**, e2440969 (2024). 39466245 10.1001/jamanetworkopen.2024.40969 PMC11519755

[33] Perlis, R. H., Goldberg, J. F., Ostacher, M. J. & Schneck, C. D. Clinical decision support for bipolar depression using large language models. *Neuropsychopharmacology* **49**, 1412–1416 (2024). 38480911 10.1038/s41386-024-01841-2 PMC11251032

[34] Priyadarshana, Y. H. P. P., Liang, Z. & Piumarta, I. ExDoRA: enhancing the transferability of large language models for depression detection using free-text explanations. *Front. Artif. Intell.* **8**, 1564828 (2025). 40469073 10.3389/frai.2025.1564828 PMC12133835

[35] Lancet Digital, T. he Health. Large language models: a new chapter in digital health. *Lancet Digit. Health* **6**, e1 (2024). 38123249 10.1016/S2589-7500(23)00254-6

[36] Heston, T. F. Safety of large language models in addressing depression. *Cureus* **15**, e50729 (2023). 10.7759/cureus.50729 PMC10727113 38111813

[37] Geijer, J., Baigi, A. & Aiff, H. Inter-rater reliability among psychiatrists when assessing depression according to the Montgomery–Åsberg depression rating scale. *Nord. J. Psychiatry* **75**, 607–613 (2021). 34156321 10.1080/08039488.2021.1918240

[38] Rai, S. et al. Key language markers of depression on social media depend on race. *Proc. Natl Acad. Sci. USA* **121**, e2319837121 (2024). 38530887 10.1073/pnas.2319837121 PMC10998627

[39] eBioMedicine Harnessing artificial intelligence for mental health care. *eBioMedicine* **111**, 105563 (2025). 39818441 10.1016/j.ebiom.2025.105563 PMC11784658

[40] Goldberg, C. B. et al. To do no harm — and the most good — with AI in health care. *Nat. Med.* **30**, 623–627 (2024). 38388841 10.1038/s41591-024-02853-7

[41] Dolev, E. L., Lutz, C. F. & Aepli, N. Does whisper understand Swiss German? An automatic, qualitative, and human evaluation. (Association for Computational Linguistics, 2024).

[42] Chan, B., Schweter, S. & Möller, T. German’s Next Language Model. In *Proc. 28th International Conference on Computational Linguistics* 6788–6796 (International Committee on Computational Linguistics, 2020).

