# Supplementary material for: Using a fine-tuned large language model for symptom-based depression evaluation
Source: NPJ Digit Med. 2025 Oct 7;8:598. doi: 10.1038/s41746-025-01982-8 (PMC12504582; doi:10.1038/s41746-025-01982-8)
Supplement: Supplementary file 1 — SupplementaryMaterial_LLM_MADRS_Weber [file 41746_2025_1982_MOESM1_ESM.pdf]

Supplementary Material

Towards Precision Psychiatry: Using a Fine-tuned Large Language Model for Symptom-based Depression Evaluation.

Samantha Weber, PhD[1,2*], Nicolas Deperrois, PhD[3], Robert Heun, MD[1,2,], Laura Frühschütz, MD[1,2,], Anna Monn, MSc[1,2], Stephanie Homan, PhD[1,4], Andrea Häfliger, MSc[1,4], Erich Seifritz, MD [1,2], Tobias Kowatsch, PhD[5,6,7], Birgit Kleim, PhD[1,4], Sebastian Olbrich, MD[1,2] & the MULTICAST consortium

Author affiliations:
1 Psychiatric University Hospital Zurich, Department of Adult Psychiatry and Psychotherapy, Psychiatric University Clinic Zurich, 8032 Zürich, Switzerland
2 Faculty of Medicine, University of Zurich, 8032 Zürich, Switzerland
3 Department of Quantitative Biomedicine, University of Zürich, 8006 Zürich, Switzerland
4 Department of Psychology, Experimental Psychopathology and Psychotherapy, University of Zurich, 8052 Zürich, Switzerland.
5 Institute for Implementation Science in Health Care, University of Zurich, 8006 Zurich, Switzerland
6 School of Medicine, University of St. Gallen, 9000 St. Gallen, Switzerland
7 Department of Management, Technology, and Economics, ETH Zurich, 8006 Zurich, Switzerland

*Correspondence:
Samantha Weber, University Hospital of Psychiatry, Zurich, Lenggstrasse 31, 8032 Zurich, Switzerland; samantha.weber@bli.uzh.ch
Sebastian Olbrich, University Hospital of Psychiatry, Zurich, Lenggstrasse 31, 8032 Zurich, Switzerland; sebastian.olbrich@bli.uzh.ch

Procedures

Within the framework of a larger study on the identification predictive markers for suicidal thoughts and behavior in a transdiagnostic cohort following discharge from inpatient psychiatric care (https://www.multicast.uzh.ch/en.html), patients underwent a full day of assessment before their discharge from the hospital. The assessment included a set of questionnaires on general health, mood and suicidal thoughts and behaviours, the Montgomery-Åsberg Depression Rating Scale (MADRS[1]) interview, a resting-state electroencephalography (EEG) measurement and an interview on positive, negative nad neutral memories, as well as autobiographical memory task[2] while under EEG, as well as an interview on intrusive memories in patients with a history of suicide attempts. Patients then received instructions for the use of an app for ecological momentary assessments (EMA[3]) after discharge. During the first and the forth week after discharge, the patients are prompted 5 times a day with a set of questionnaires to answer. Patients returned to a follow-up visits after the EMA data collection has been completed. During the follow-up visit, the baseline study procedures were repeated.

Demographic Information

The patients interviewed included 27 females, 15 males, and 2 non-binary individuals, with a mean age of 36 years. Individual diagnoses, age, and gender are provided in Supplementary Table 1, while individual medication details are listed in Supplementary Table 2.

Supplementary Table 1. Age, gender and diagnoses of individual patients from the dataset.

ID	Age	Gender	Diag1	Diag2	Diag3	Diag4	Diag5	Diag6	Diag7	Diag8	Diag9
**1**	26	m	F61	F12.1							
**2**	52	f	F33.2	F60.31	F10.2						
**3**	26	f	F43.1								
**4**	63	f	F33.2								
**5**	46	m	F33.2								
**6**	25	f	F33.1								
**7**	31	m	F33.2	F60.31							
**8**	47	f	F33.2	F43.1							
**9**	33	m	F33.2	F15.2	F13.2	F10.1	F14.1	F90.0	F43.1	F50.9	F17.2
**10**	27	f	F32.2	F42.0	Z73						
**11**	23	f	F10.2	F10.3	F12.1	F33.2					
**12**	27	m	F32.2	F65.4	F98.88	F90.0	F84.5				
**13**	49	m	F33.2	F41.1.	F61						
**14**	45	f	F33.1	G35.9							
**15**	22	m	F32.3	F90.0	F43.1						
**16**	25	f	F33.2	F60.31	F90.0						
**17**	41	m	F33.0								
**18**	52	f	F43.1								
**19**	30	m	F33.3	F42.2	F84.5						
**20**	47	f	F33.2								
**21**	54	m									
**22**	23	f	F33.2	F43.1	F61	F40.1					
**23**	25	f	F33.2.	F42.2							
**24**	24	non-binary	F33.1								

(Continuation) Supplementary Table 1. Age, gender and diagnoses of individual patients from the dataset.

**25**	54	f	F33.								
**26**	19	f	F33.1	Z73	F45.40						
**27**	23	non-binary	F43.1	F60.31	F90.0						
**28**	31	f	F31.4	F60.5	F84.5						
**29**	39	m	F33.1	F60.31	F10.1	F12.1	F17.1				
**30**	21	f	F13.2	F60.31	F33.2	F12.2	F90.0				
**31**	30	f	F33.2	F50.0	F10.1						
**32**	56	f	F33.1	G81.1	G40.1						
**33**	32	m	F25.1	F90.0	F10.1						
**34**	20	f	F90.0	F60.31							
**35**	54	f	F32.2	F10.0	F10.2						
**36**	30	f	F43.1	F32.1	F90.0						
**37**	28	f	F31.3								
**38**	52	m	F33.2	F60.8							
**39**	64	m	F33.2	F61	F14.2	F11.2					
**40**	56	f	F33.1	F13.0	Z73	F90.0	M54.86				
**41**	28	f									
**42**	24	m									
**43**	25	f	6B41 (cPTSD)	F33.1							
**44**	35	f	F43.1	F90.0	F12.7	F10.1					

Supplementary Table 2. Medication of individual patients from the dataset. IDs with *a* and *b* correspond to the different timepoints with *a* = Baseline measurement and *b* = follow-up measurement.

ID	Med	Dos1	Med2	Dos2	Med3	Dos3	Med4	Dos4	Med5	Dos5	Med6
1a	Nihil										
1b	Nihil										
2a	Etiltox	200mg	Sequase Ret.	50mg	Sertalin	150mg					
2b	Zoloft	200mg	Sequase	smallest dose	Elitox	1 Pill	Metamizol	1TL			
3	Trittico	50mg									
4a	Sertralin	200mg									
4b	Sertralin	200mg									
5a	Duloxetin	90mg									
5b	Duloxetin	60 mg									
6	Venlafaxin Ret.	225mg									
7	Nihil										
8	Nihil										
9a	Concerta ret.	90mg	Trittico	250mg	Venlafaxin	300mg					
9b	Concerta	90mg	Trittico	250mg	Venlafaxin	300mg					
10	Cipralex	20mg	Aripiprazol Mepha	15 mg							
11a	Cipralex	15mg	Sequase	25mg		0					
11b	Circaplex	10mg	Sequase on demand	25 mg	Vitamin B12	1 Pill					
12a	Quilonorm ret.	12.2 mmol	Sequase	100 mg							
12b	Ritalin	40mg	Oxidan	20mg							
13a	Venlafaxin Ret.	112,5 mg									
13b	Venalafaxin Ret.	112,5 mg	Sequase	on demand							
14a	Cipralex	15mg	Escitalopram	15mg							
14b	Escitalopram	10mg									
15	Cipralex	20mg	Trittico	50mg							
16	Cipralex	30 mg	Concerta	36mg	Trittico Ret.	150mg	Trittico	100mg			

(Continuation) Supplementary Table 2. Medication of individual patients from the dataset. IDs with *a* and *b* correspond to the different timepoints with *a* = Baseline measurement and *b* = follow-up measurement.

ID	Med	Dos1	Med2	Dos2	Med3	Dos3	Med4	Dos4	Med5	Dos5	Med6
17	Trittico Ret.	50 mg									
18a	Aripiprazol	10mg	Selincro	54mg	Sequase XR Ret	150mg	Trittico Ret	150mg	Trittico	150mg	Euthyrox
18b	Aripiprazol	10mg	Selincro	54mg	Sequase XR Ret	150mg	Trittico Ret	150mg	Trittico	150mg	Euthyrox
19	Olanzapin	5mg	Reagila	4.5mg	Sertralin	250 mg	Trittico	50 mg			
20	Trittico Ret.	49.5mg	Trittico	50 mg	Velnafaxin Ret.	150mg					
21a	Fluoxetin	60mg	Olanzapin	10mg							
21b	Fluoxetin	60mg	Olanzapin	10mg							
22a	Aripiprazol	5mg	Duloxetin	90mg	Tretinac	5mg					
22b	Aripiprazol	5mg	Duloxetin	90mg	Tretinac	5mg					
23a	Sequase	25mg	Sertralin	150mg	Surmontil	50mg					
23b	Sertralin	150mg									
24	Sequase XR Ret	100mg	Sertralin	100mg							
25a	Cipralex	10mg	Trittico	50mg							
25b	Cipralex	10mg									
26	Fluoxetin	30mg									
27a	Ritalin	20mg									
27b	Ritalin	20mg									
28a	Lamictal	250mg	Sertralin	150mg	Trittico	150mg					
28b	Lamictal	300mg	Sertralin	150mg	Trittico	50mg					
29a	Sequase	50mg	Venlafaxin	150mg	Redormin	250mg					
29b	Sequase	50mg	Venlafaxin	150mg	Redormin	250mg					
30	Duloxetin	60mg	Lamictal	50mg	Trittico	50mg	Redormin	500mg	Xanax	1mg	
31a	Redormin	500mg	Sequase	25mg	Sertralin	150mg					
31b	Sequase	25mg	Sertralin	150mg							
32	Redormin	500mg	Neurontin	400mg	Trittico	50mg	Venlafaxin	225mg			
33a	Aripiprazol	15mg	Brintellix	20mg	Elvanse	40mg	Lamictal	200mg	Quilonorm	18,3mmol	Sequase
33b	Aripiprazol	15mg	Brintellix	20mg	Elvanse	40mg	Lamictal	200mg	Lithium (Quilonorm)	18.3mmol	

(Continuation) Supplementary Table 2. Medication of individual patients from the dataset. IDs with *a* and *b* correspond to the different timepoints with *a* = Baseline measurement and *b* = follow-up measurement.

ID	Med	Dos1	Med2	Dos2	Med3	Dos3	Med4	Dos4	Med5	Dos5	Med6
34	Concerta	18mg									
35a	CIPRALEX	20mg	Mirtazapin	15mg							
35b	CIPRALEX	30mg									
36	Nihil										
37	Lithium	450 mg									
38	Duloxetin	90mg	Trittico	350mg							
39a	Ketamin nasal	34.29 mg	Dipiperon	40mg	Sequase	25mg	Cipralex	10mg	Subutex	8mg	
39b	Ketamin nasal	34.29 mg	Dipiperon	40mg	Sequase	25mg	Cipralex	10mg	Subutex	8mg	
40	Venlafaxin Viatris ER Ret	150mg	Elvanse	50mg	Trittico	50mg	Calcimagon D3 500/800				
41	Nihil										
42	Nihil										
43	Nihil										
44	ARIPIPRAZOL	25mg	VALDOXAN	50mg	WELLBUTRIN	300mg					

MADRS Interview

Instructions translated from the German version that were used by the investigators.

Supplementary Table 3. MADRS Interview Instructions.

Topic	Instructions	Scoring
0: Apparent sadness	This item includes despondency, dejection, and despair expressed through speech, facial expression, and posture. Assess based on severity and the inability to be cheered up.	0: No sadness.<br>1:<br>2: Appears dejected but can be cheered up easily.<br>3:<br>4: Seems sad and unhappy most of the time.<br>5:<br>6: Looks sad and unhappy all the time. Extreme dejection.
2: Inner tension	Includes the patient's description of a depressed mood, whether visible or not, including discouragement, dejection, feelings of helplessness, and hopelessness. Assess based on severity, duration, and ability to be influenced by external events.	0: Occasional sadness appropriate to circumstances.<br>1:<br>2: Feels sad or dejected but can be cheered up easily.<br>3:<br>4: Constant sadness and gloom but still influenced by external circumstances.<br>5:<br>6: Persistent, unchanging sadness, dejection, or hopelessness.
3: Sleep disturbances	Includes a vague sense of discomfort, irritability, restlessness, inner excitement up to anxiety and panic. Assess based on severity, frequency, duration, and extent of seeking reassurance.	0: Sleeps as usual.<br>1:<br>2: Mild difficulty falling asleep. Superficial, restless sleep. Slightly reduced sleep duration.<br>3:<br>4: Sleep reduced or interrupted by at least 2 hours.<br>5:<br>6: Sleeps less than 2-3 hours.
4: Loss of appetite	Includes the feeling of having less appetite compared to normal. Assess based on the severity of appetite loss or how much one has to force themselves to eat.	0: Normal or increased appetite.<br>1:<br>2: Slightly reduced appetite.<br>3:<br>4: No appetite. Food does not taste good.<br>5:<br>6: Must be persuaded to eat.
5: Difficulties concentrating	Includes difficulties in concentrating, ranging from simple trouble gathering thoughts to a complete inability to focus. Assess based on severity, frequency, and extent of the impairment.	0: No concentration difficulties.<br>1:<br>2: Occasional trouble gathering thoughts.<br>3:<br>4: Difficulty concentrating and holding a thought. Affects reading or conversations.<br>5:<br>6: Unable to read or hold a conversation without difficulty.
6: Lassitude	Includes difficulties in initiating activities or sluggishness in starting and completing everyday tasks.	0: Almost no difficulty starting activities. No sluggishness.<br>1:<br>2: Difficulty starting an activity.<br>3:<br>4: Trouble starting simple routine activities, completing them only with effort.<br>5:<br>6: Complete lack of initiative. Unable to do anything without assistance.
7: Emotional numbness	Includes a subjective feeling of reduced interest in surroundings or activities that previously brought joy. The ability to respond to circumstances or other people with appropriate feelings is diminished.	0: Normal interest in surroundings or other people.<br>1:<br>2: Less enjoyment in past interests.<br>3:<br>4: Loss of interest in surroundings. Loss of feelings for friends and acquaintances.<br>5:<br>6: Total emotional numbness. Unable to feel anger, sadness, or joy. Complete or painfully perceived loss of emotions for close relatives and friends.
8: Pessimistic thoughts	Includes feelings of guilt, worthlessness, self-reproach, sinfulness, remorse, and doom.	0: No pessimistic thoughts.<br>1:<br>2: Occasional thoughts of failure, self-reproach, and self-degradation.<br>3:<br>4: Persistent self-accusations. Clear but still logically reasonable ideas of guilt and sin. Increasing pessimism about the future.<br>5:<br>6: Delusions of ruin, feelings of remorse, or irredeemable sins. Self-accusations that are irrational yet unshakable.
9: Suicidal ideations	Includes the feeling that life is not worth living, that natural death would be a relief, suicidal thoughts, and	0: Enjoys life or believes that life must be taken as it comes.<br>1:<br>2: Occasionally feels life is not worth living.

	preparations for suicide. Suicide attempts should not directly influence the rating.	3:<br>4: Would rather be dead. Frequent suicidal thoughts. Suicide is seen as a possible way out, but no specific plans or intentions.<br>5:<br>6: Clear suicidal plans when an opportunity arises. Active preparation for suicide.

Scoring and Interpretation

Each item is rated on a **0-6 scale**, with **a total score ranging from 0 to 60**. The higher the total score, the more severe the depression.

- **0-6:** No or minimal depression
- **7-19:** Mild depression
- **20-34:** Moderate depression
- **35-60:** Severe depression

Synthetic Data Generation

We applied a pre-trained Sentence-BERT model (https://huggingface.co/sentence-transformers/paraphrase-MiniLM-L6-v2) to embed the transcriptions of real patient interviews and synthetic data. These embeddings were compared using cosine similarity to assess how closely the synthetic sentences align with the real ones.

The average cosine similarity between the real and synthetic data was 0.61, indicating moderate similarity. The highest similarity was 1, suggesting high similarity between some of the real and synthetic data pairs. The lowest similarity was -0.12, indicating that some synthetic sentences strongly differed from the real data. This suggests that the synthetic data captures a reasonable amount of content similarity to the real data, but it also includes cases where the synthetic sentences strongly differ, likely due to the synthetic data covering extreme severity cases that may not be fully represented in the real dataset.

Training and Evaluation *MADRS-BERT*

Supplementary Figure 1. (A) Training Loss and (B) Validation Loss.

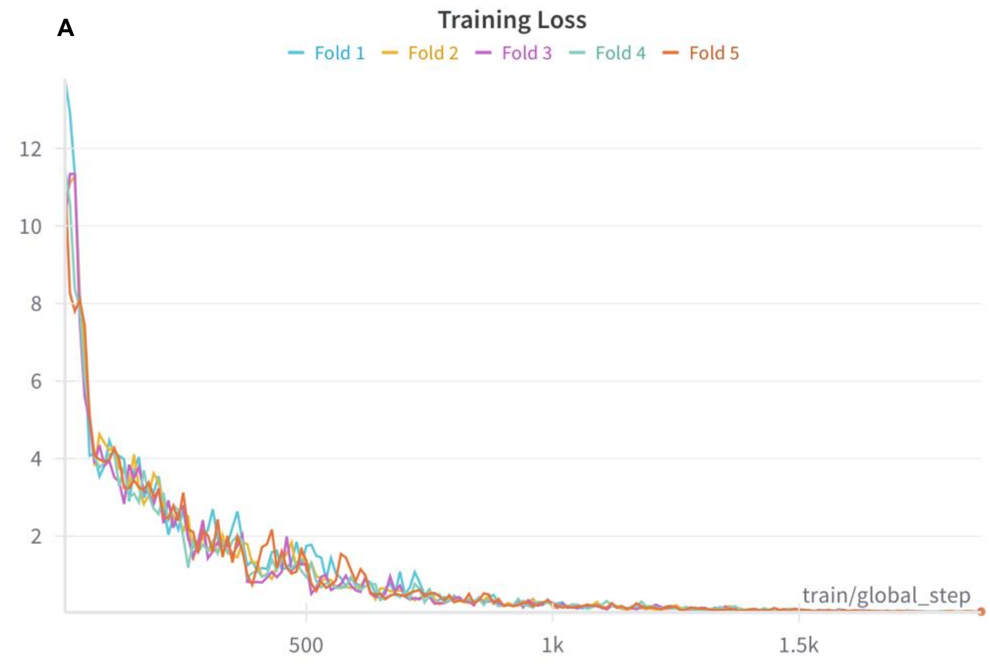

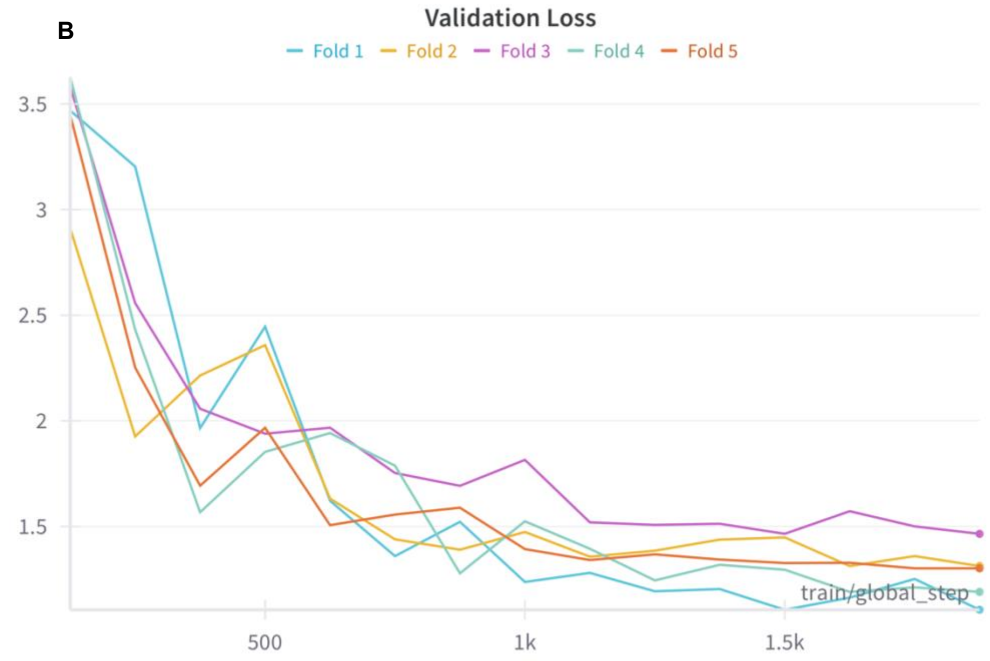

Supplementary Figure 2. (A) Validation Mean Squared Error (MSE), (B) Validation Mean Absolute Error (MAE), (C) Validation Accuracy strict, and (D) Validation Accuracy flexible

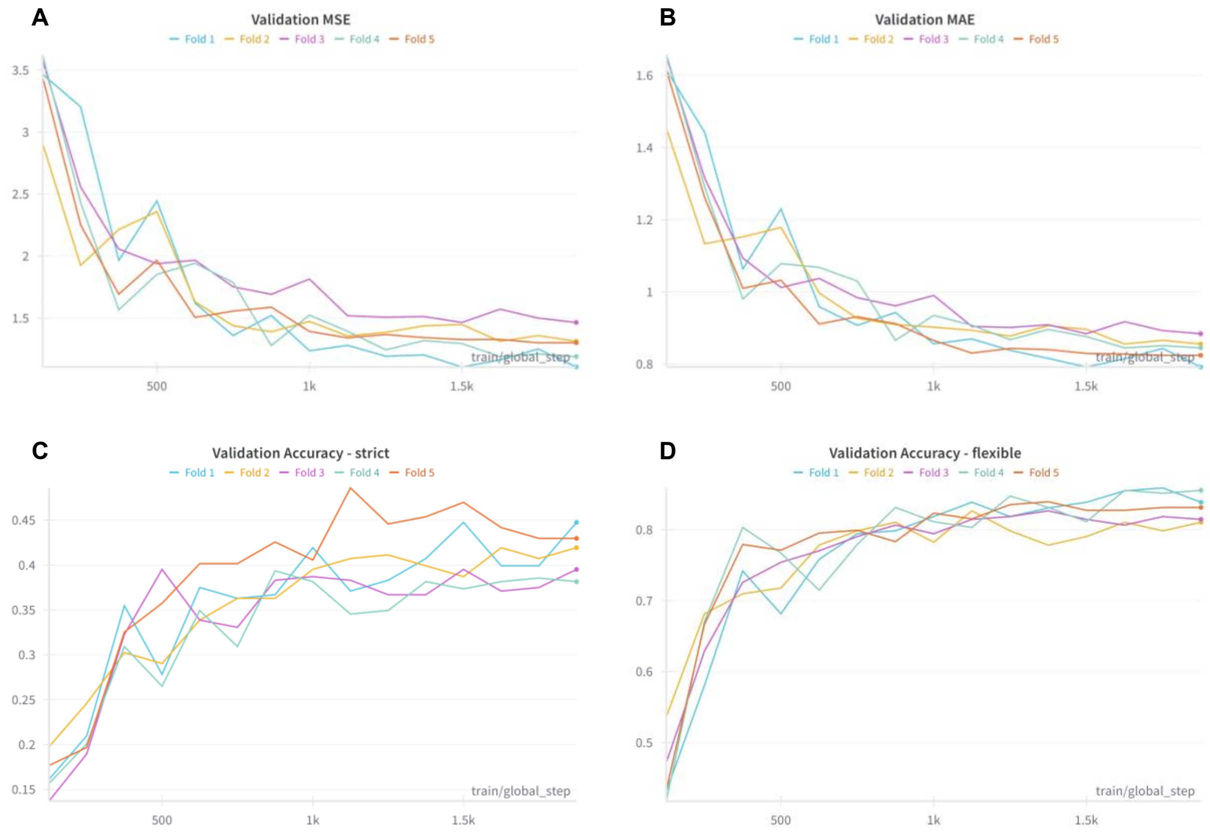

Supplementary Figure 3. Learning curves for nine MADRS topics under the strict accuracy criterion. Each line corresponds to one MADRS item. For each outer cross-validation fold, models were trained on increasing fractions (5–80%) of the entire dataset, with evaluation always performed on a fixed validation set comprising 20% of the full dataset. The x-axis indicates the proportion of the full dataset used for training, while the y-axis shows the mean strict accuracy across outer folds. Error bars indicate the standard error of the mean (SEM) across folds.

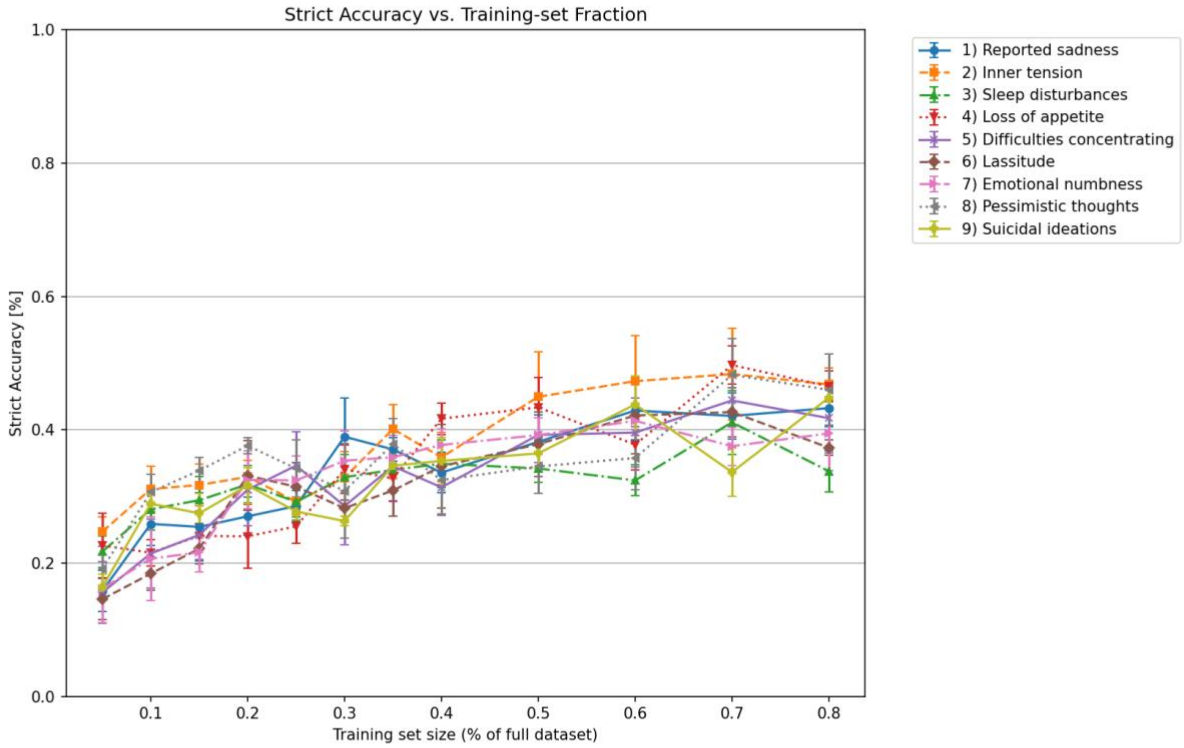

Classification Performance BERT-base and BERT-base-flexible

Supplementary Figure 4. Confusion matrices for *BERT-base* model. Each confusion matrix shows prediction performance for a single MADRS item, comparing the predicted (x-axis) versus the actual (y-axis) MADRS scores. Values reflect the number of individual item-level segments across all test folds. The colour intensity represents the count of predictions, with darker shades indicating higher values. Diagonale entries represent correctly classified instances, while off-diagonal entries indicate errors.

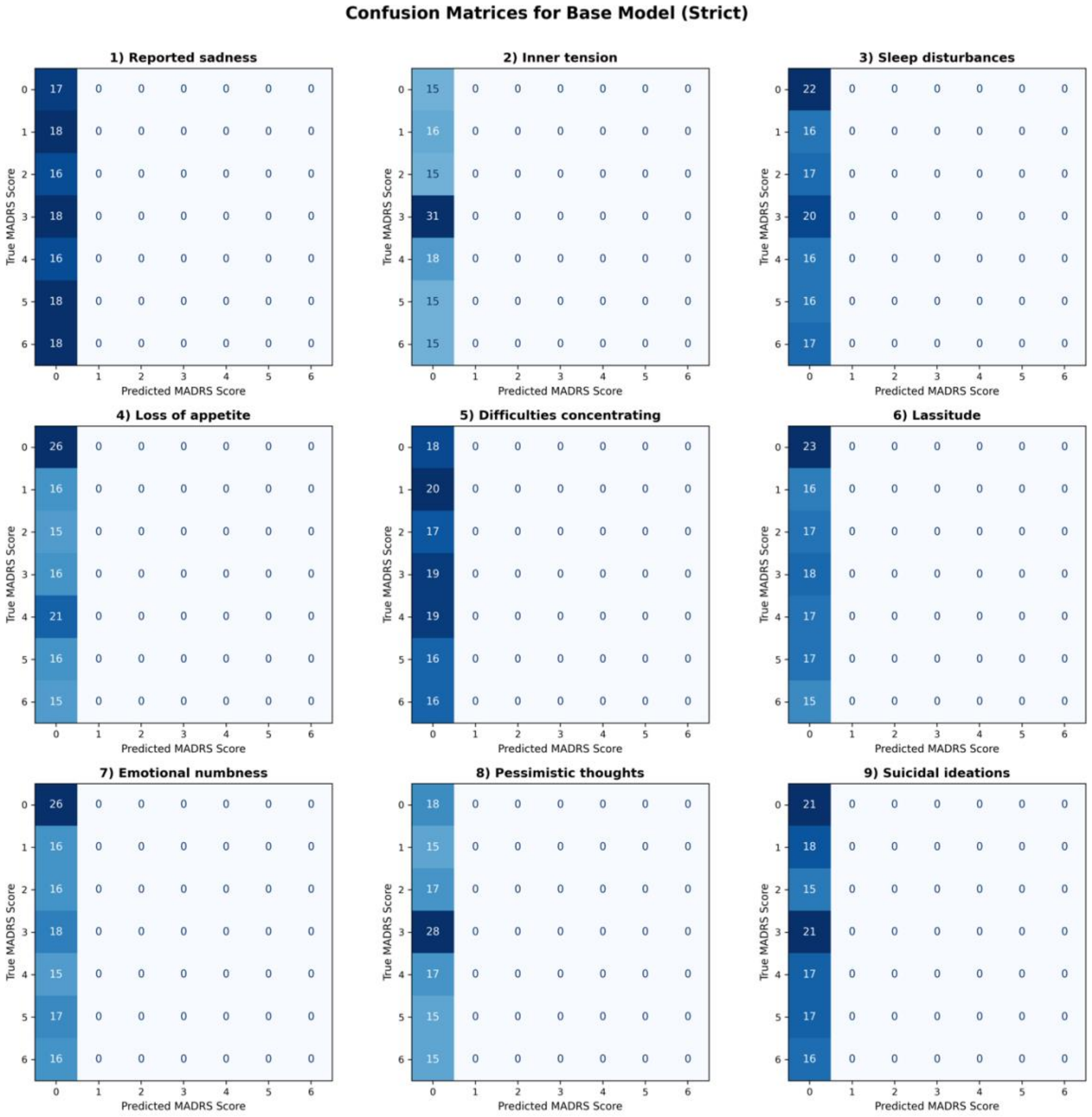

Supplementary Figure 5. Confusion matrices for *BERT-base-flexible* model. Each confusion matrix shows prediction performance for a single MADRS item, comparing the predicted (x-axis) versus the actual (y-axis) MADRS scores. Values reflect the number of individual item-level segments across all test folds. The colour intensity represents the count of predictions, with darker shades indicating higher values. Diagonale entries represent correctly classified scores, while off-diagonal entries indicate errors. The model's performance is shown under the flexible criteria, with predictions within ±1 of the true label considered as a correct prediction.

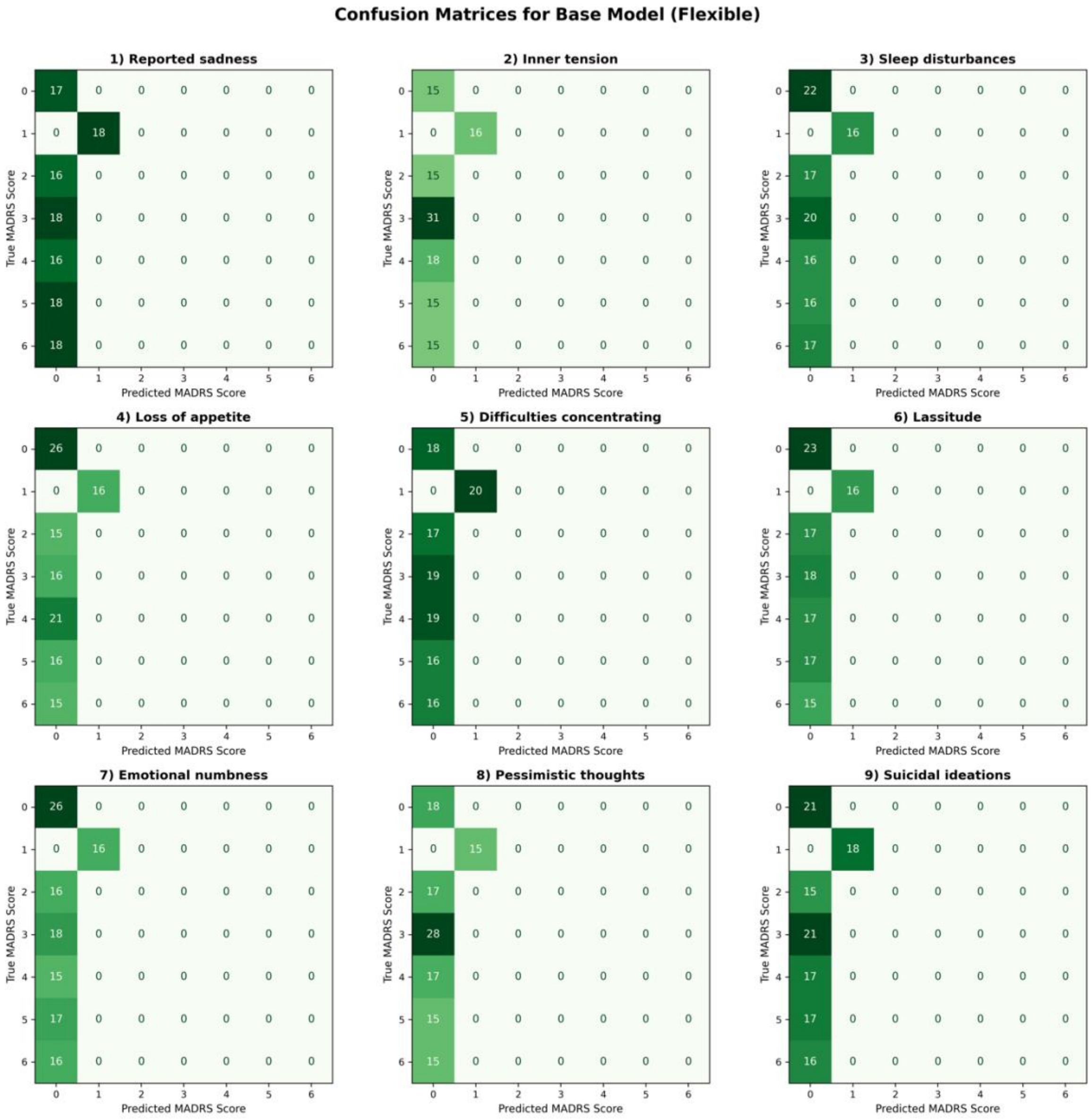

Supplementary Table 4. Comparison of *BERT-base* and Baseline Predictor (Mean Regression Model) Performance Across MADRS items. The table reports the Mean Score and Mean Absolute Error (MAE) for the baseline predictor and the base model (*BERT-base*) across all nine MADRS items. The baseline predictor assigns the mean MADRS score per topic as the predicted value, serving as a naive statistical reference. MAE quantifies the prediction error, with lower values indicating better performance. Bold numbers highlight the best results across.

MADRS Item	Mean MADRS Score	MAE ↓ (±std)	
		**Baseline**	***BERT-base***
Reported sadness	3.0	1.7	3.0 (±0.24)
Inner tension	3.0	1.5	2.9 (±0.44)
Sleep disturbances	2.9	1.7	2.8 (±0.34)
Loss of appetite	2.8	1.8	2.7 (±0.32)
Difficulties concentrating	2.9	1.7	2.8 (±0.36)
Lassitude	2.8	1.8	2.8 (±0.37)
Emotional numbness	2.8	1.8	2.7 (±0.49)
Pessimistic thoughts	2.9	1.6	2.8 (±0.38)
Suicidal ideations	2.9	1.7	2.8 (±0.16)

Statistical evaluation

When comparing the classification performance between the fine-tuned (*MADRS-BERT*) and base models (*BERT-base*) under strict and flexible evaluation criteria, McNemar's test for statistical significance showed significantly better accuracy of the 1) *MADRS-BERT-flexible* versus *BERT-base-flexible* across all items ($P < 0.0001$). Likewise, 2) *MADRS-BERT* performed better across all items than *BERT-base* ($P < 0.0001$). These results highlight that fine-tuning significantly improves classification performance under flexible and strict conditions. Moreover, 3) *MADRS-BERT-flexible* performed better across all items than *MADRS-BERT* ($P < 0.0001$), and 4) *BERT-base-flexible* performed better across all items than *BERT-base* ($P < 0.0001$), highlighting that classification performance improves under flexible criteria independently of the model. The contingency tables and results per item can be found in Supplementary Figures 6-9.

Supplementary Figure 6. Contingency tables comparing *MADRS-BERT* versus *BERT-base* model outcomes across topics. Each table shows the counts of outcomes classified as where the models where correct versus not correct. The y-axis indicates the outcomes of *BERT-base*, while the x-axis represents the outcomes of *MADRS-BERT*.

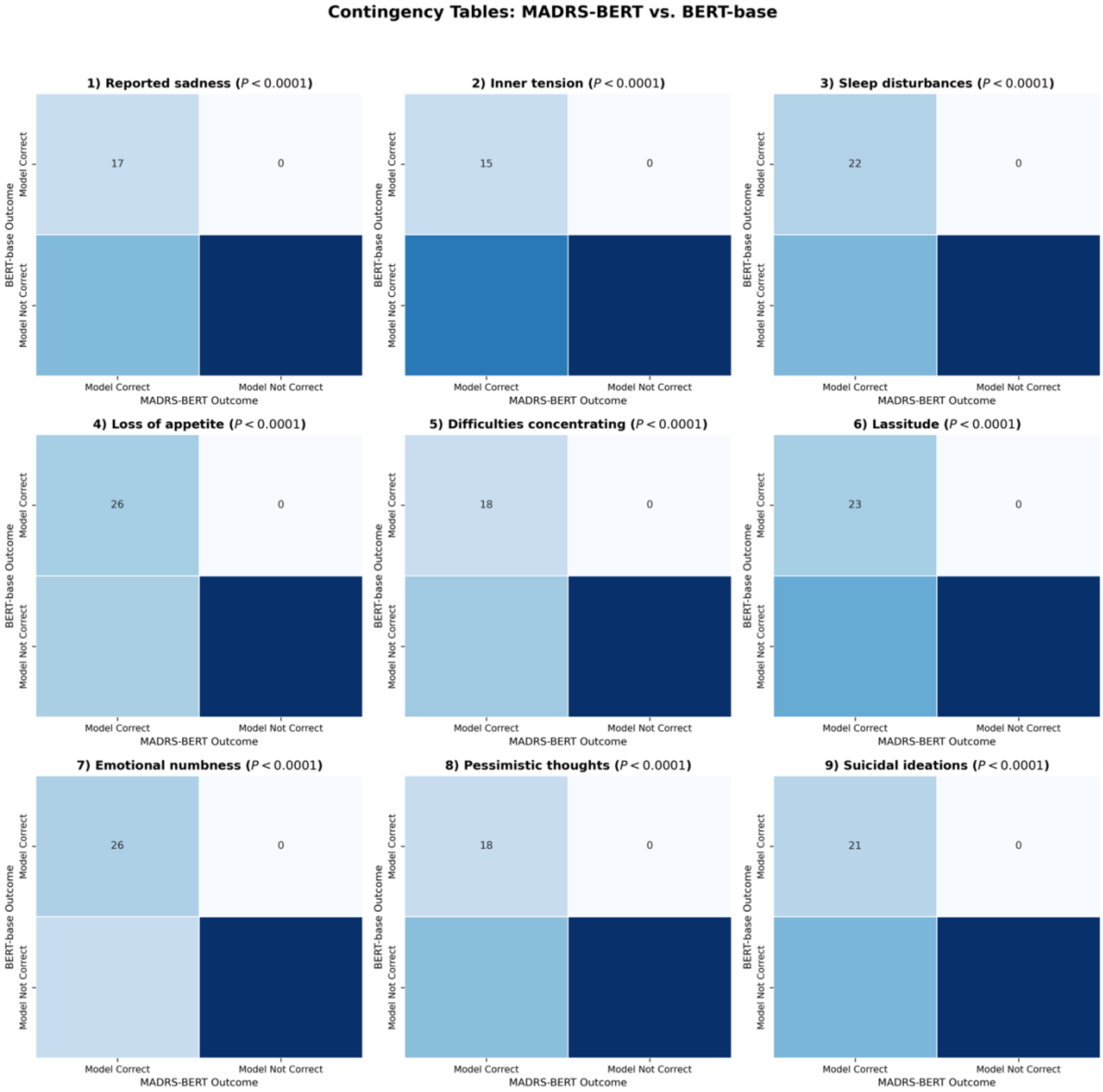

Supplementary Figure 7. Contingency tables comparing *MADRS-BERT-flexible* versus *MADRS-BERT* model outcomes across topics. Each table shows the counts of outcomes classified as where the models where correct versus not correct. The y-axis indicates the outcomes of *MADRS-BERT*, while the x-axis represents the outcomes of *MADRS-BERT-flexible*.

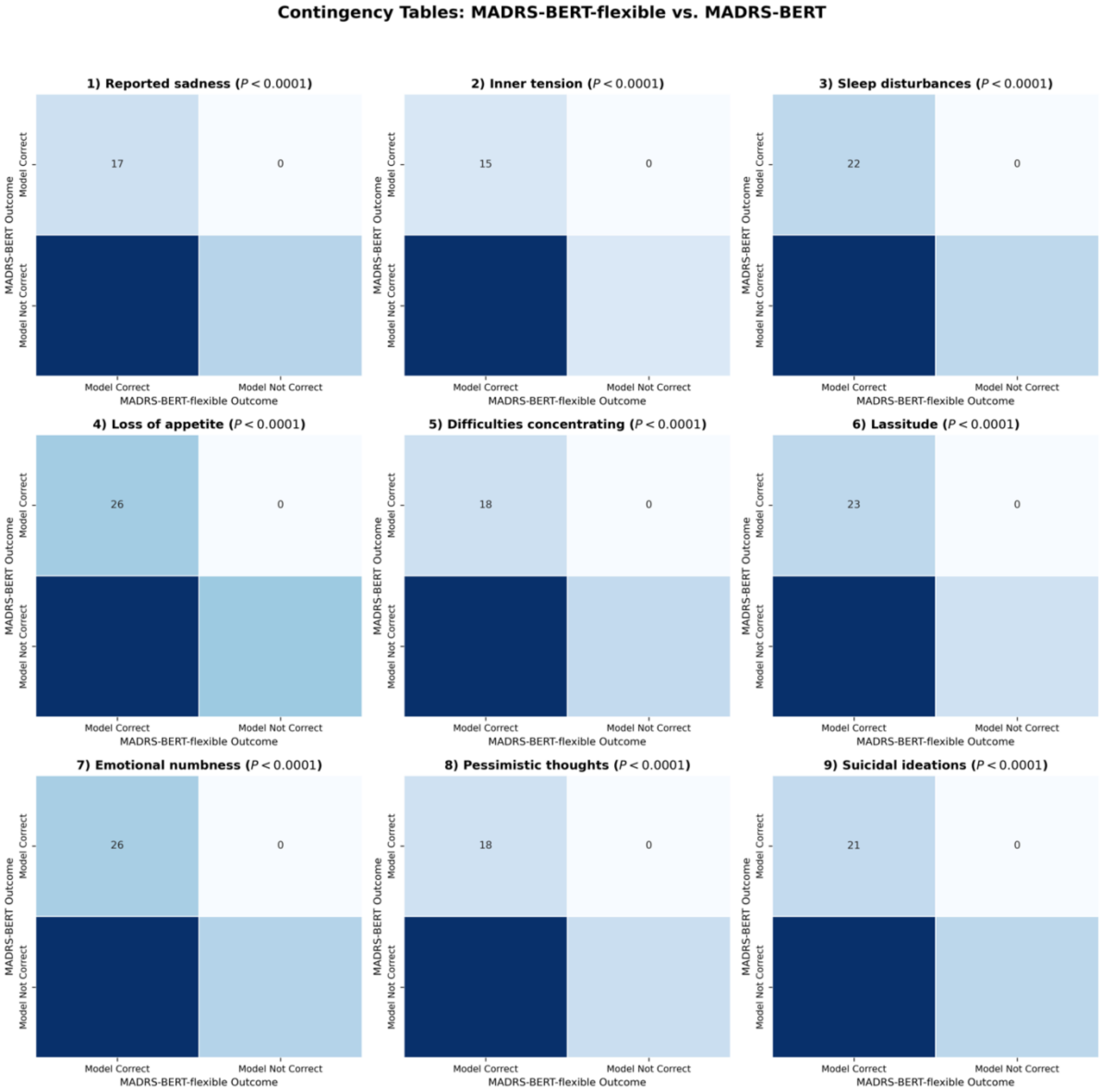

Supplementary Figure 8. Contingency tables comparing *MADRS-BERT- flexible* versus *BERT-base-flexible* model outcomes across topics. Each table shows the counts of outcomes classified as where the models where correct versus not correct. The y-axis indicates the outcomes of *BERT-base-flexible*, while the x-axis represents the outcomes of *MADRS-BERT-flexible*.

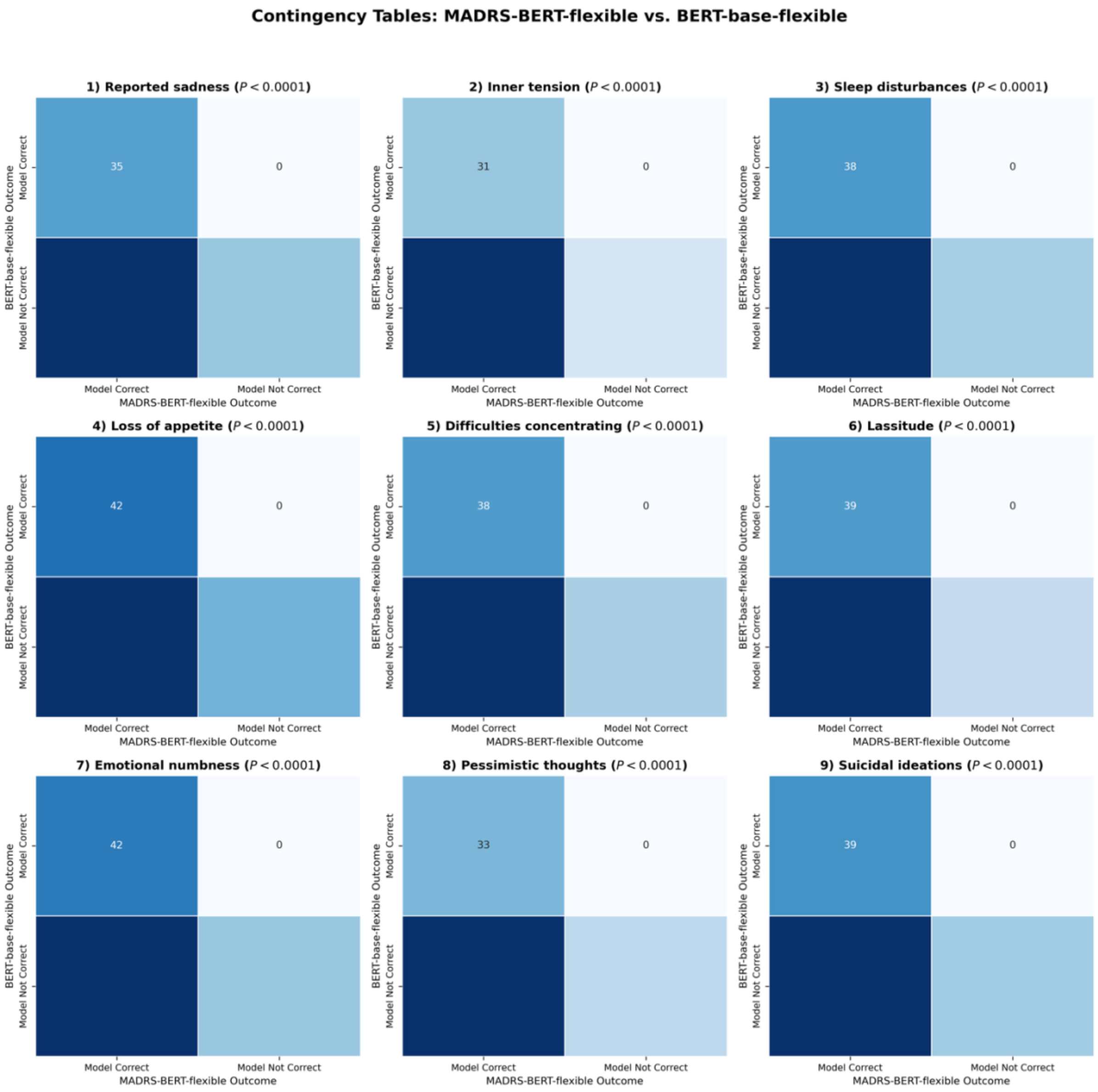

Supplementary Figure 9. Contingency tables comparing *BERT-base-flexible* versus *BERT-base* model outcomes across topics. Each table shows the counts of outcomes classified as where the models where correct versus not correct. The y-axis indicates the outcomes of *BERT-base*, while the x-axis represents the outcomes of *BERT-base-flexible*.

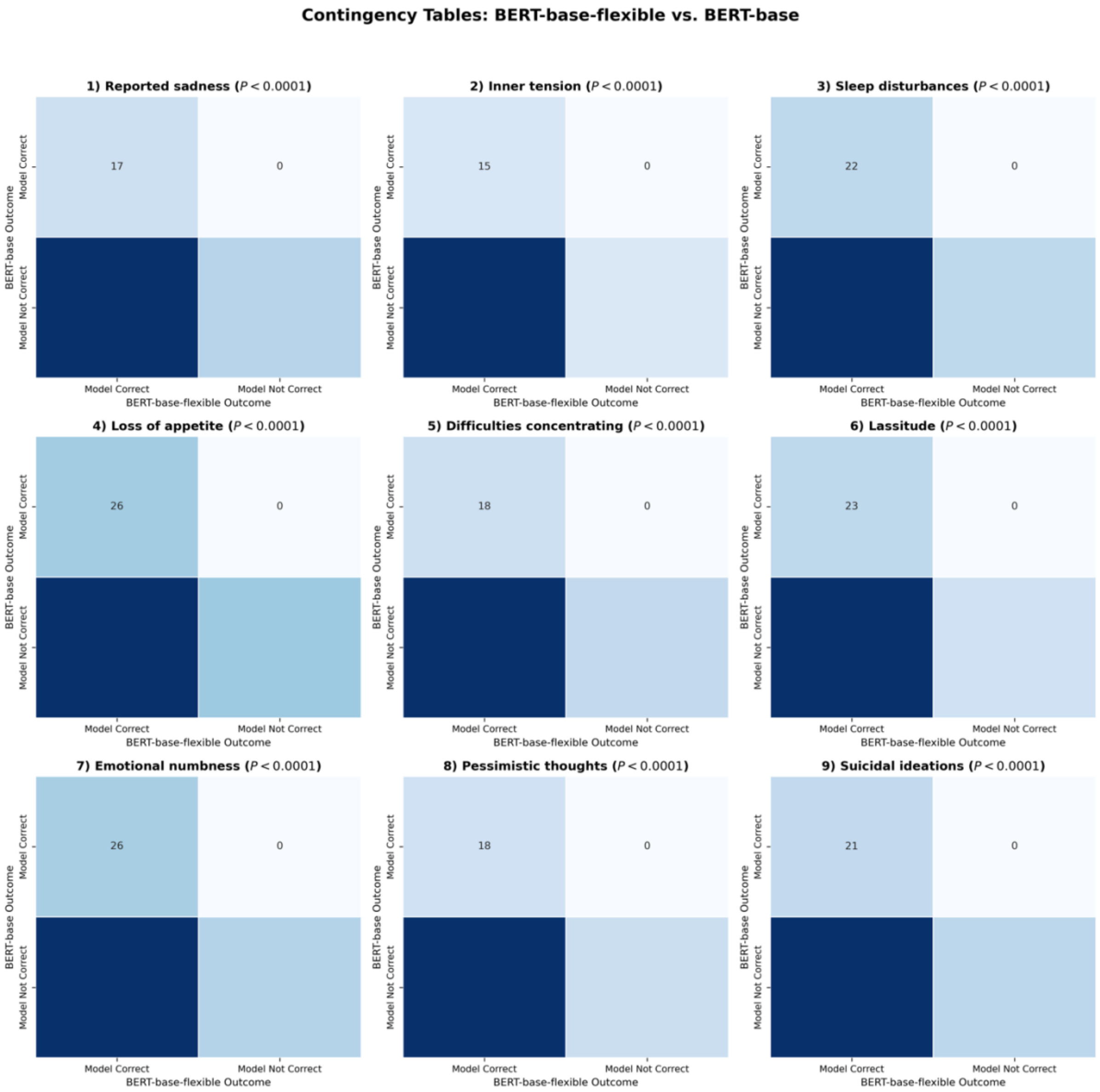

Classification Performance MADRS-BERT-base and MADRS-BERT-flexible using real data only

Supplementary Figure 10. Confusion matrices for *MADRS-BERT* model using real data only (*N*=64 interviews). Each confusion matrix shows prediction performance for a single MADRS item, comparing the predicted (x-axis) versus the actual (y-axis) MADRS scores. Values reflect the number of individual item-level segments across all test folds. The colour intensity represents the count of predictions, with darker shades indicating higher values. Diagonale entries represent correctly classified instances, while off-diagonal entries indicate errors.

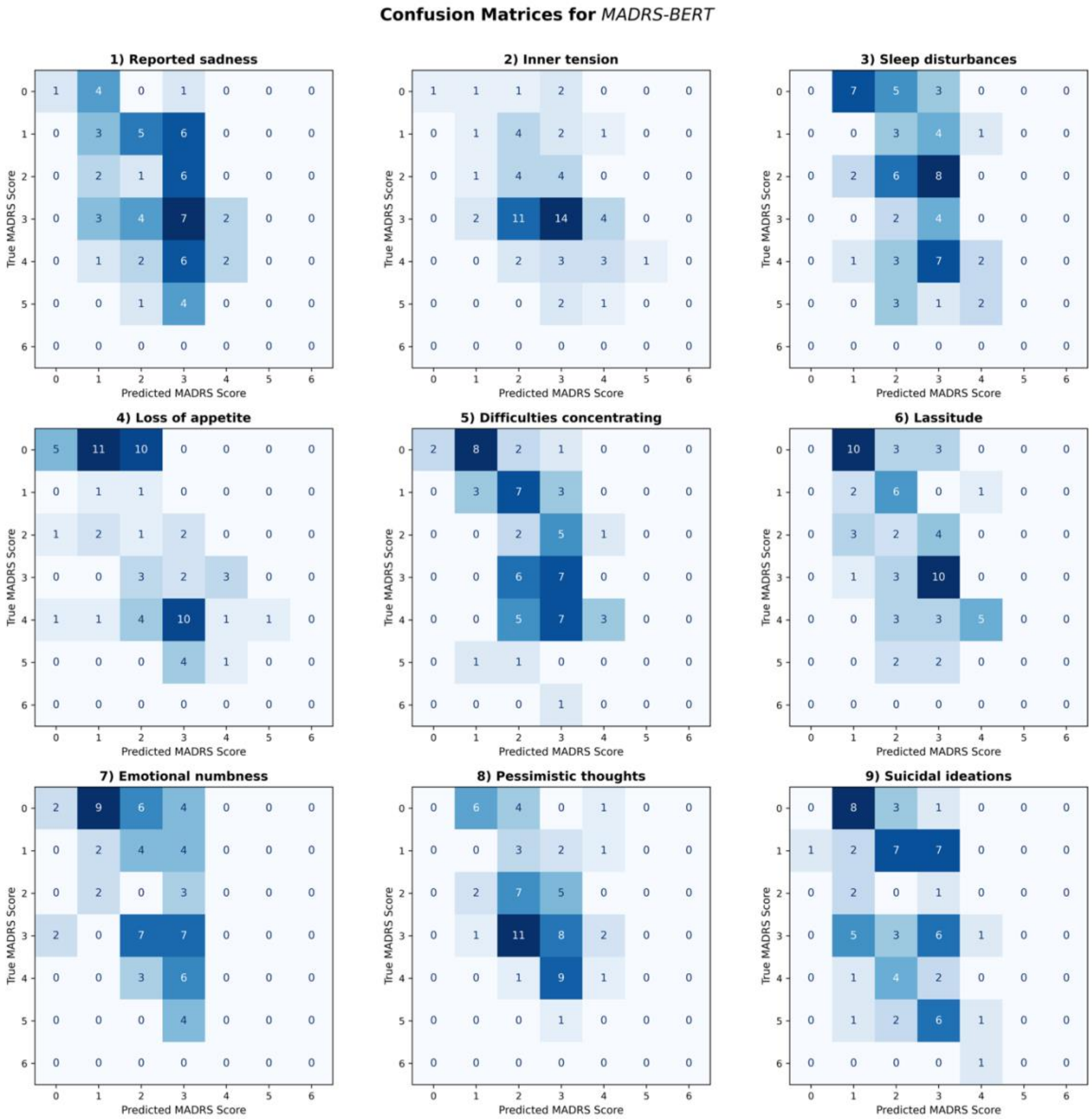

Supplementary Figure 11. Confusion matrices for *MADRS-BERT-flexible* model using real data only (*N*=64 interviews). Each confusion matrix shows prediction performance for a single MADRS item, comparing the predicted (x-axis) versus the actual (y-axis) MADRS scores. Values reflect the number of individual item-level segments across all test folds. The colour intensity represents the count of predictions, with darker shades indicating higher values. Diagonale entries represent correctly classified scores, while off-diagonal entries indicate errors. The model's performance is shown under the flexible criteria, with predictions within ±1 of the true label considered as a correct prediction.

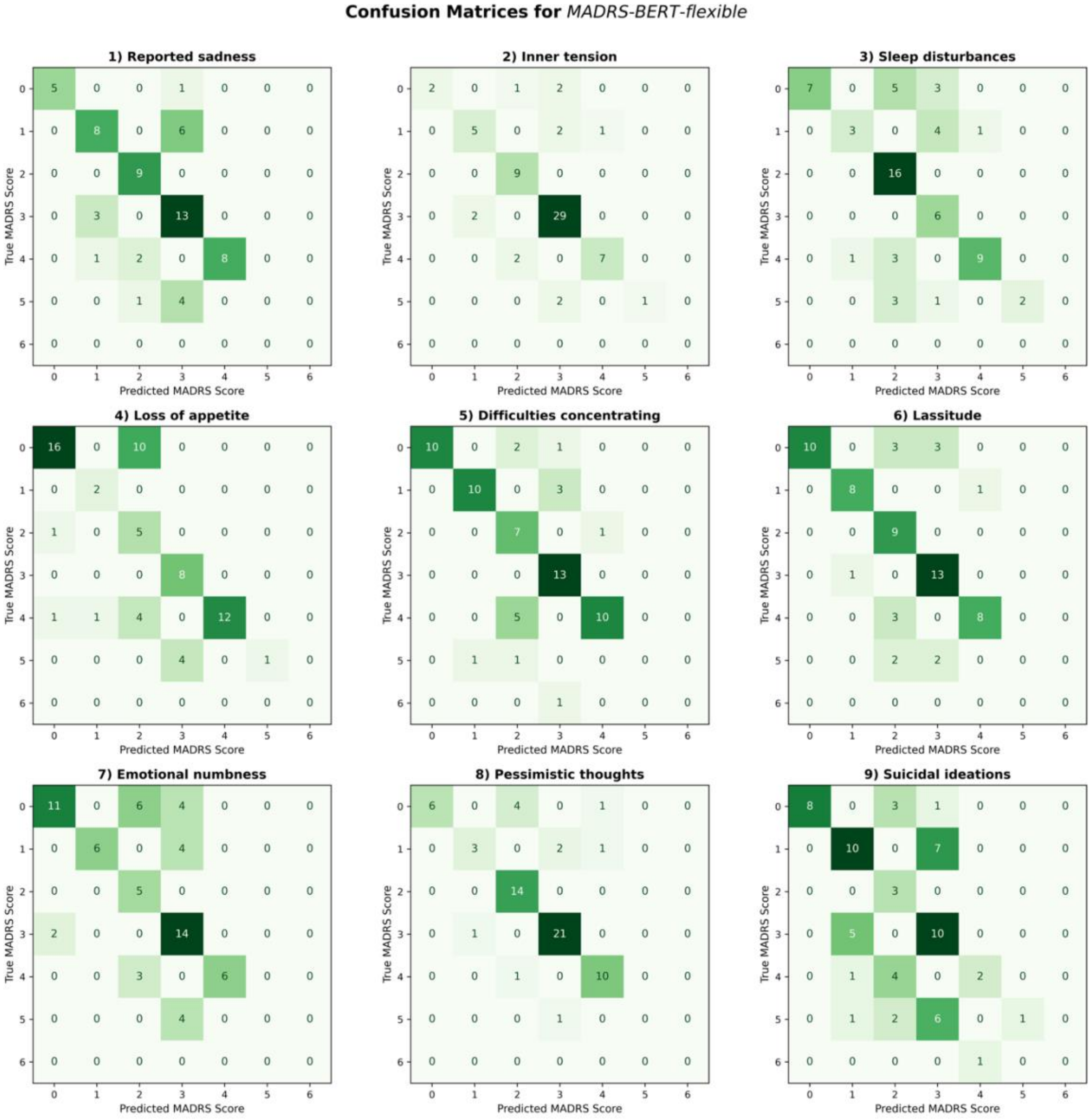

Supplementary Table 5. Comparison of *MADRS-BERT* and Baseline Predictor (Mean Regression Model) Performance Across MADRS items using real data only (*N*=64 interviews). The table reports the Mean Score and Mean Absolute Error (MAE) for the baseline predictor and the base model (*MADRS-BERT*) across all nine MADRS items. The baseline predictor assigns the mean MADRS score per topic as the predicted value, serving as a naive statistical reference. MAE quantifies the prediction error, with lower values indicating better performance. Bold numbers highlight the best results across.

MADRS Item	Mean MADRS Score	MAE ↓ (±std)	
		Baseline	*MADRS-BERT*
Reported sadness	2.4	1.3	1.1 (±0.21)
Inner tension	2.6	1.0	0.9 (±0.20)
Sleep disturbances	2.2	1.4	1.3 (±0.21)
Loss of appetite	2.1	1.7	1.2 (±0.14)
Difficulties concentrating	2.2	1.4	1.0 (±0.24)
Lassitude	2.1	1.4	1.1 (±0.35)
Emotional numbness	1.9	1.5	1.3 (±0.11)
Pessimistic thoughts	2.3	1.1	1.0 (±0.08)
Suicidal ideations	2.3	1.6	1.4 (±0.04)

STARD Checklist

Section & Topic	No	Item	Reported on page #
**TITLE OR ABSTRACT**			
	**1**	Identification as a study of diagnostic accuracy using at least one measure of accuracy (such as sensitivity, specificity, predictive values, or AUC)	1,2
**ABSTRACT**			
	**2**	Structured summary of study design, methods, results, and conclusions (for specific guidance, see STARD for Abstracts)	2
**INTRODUCTION**			
	**3**	Scientific and clinical background, including the intended use and clinical role of the index test	3
	**4**	Study objectives and hypotheses	3
**METHODS**			
Study design	**5**	Whether data collection was planned before the index test and reference standard were performed (prospective study) or after (retrospective study)	10
Participants	**6**	Eligibility criteria	10
	**7**	On what basis potentially eligible participants were identified (such as symptoms, results from previous tests, inclusion in registry)	10
	**8**	Where and when potentially eligible participants were identified (setting, location and dates)	10
	**9**	Whether participants formed a consecutive, random or convenience series	10
Test methods	**10a**	Index test, in sufficient detail to allow replication	11-14
	**10b**	Reference standard, in sufficient detail to allow replication	12-14
	**11**	Rationale for choosing the reference standard (if alternatives exist)	12-14
	**12a**	Definition of and rationale for test positivity cut-offs or result categories of the index test, distinguishing pre-specified from exploratory	13-14
	**12b**	Definition of and rationale for test positivity cut-offs or result categories of the reference standard, distinguishing pre-specified from exploratory	13-14
	**13a**	Whether clinical information and reference standard results were available to the performers/readers of the index test	14
	**13b**	Whether clinical information and index test results were available to the assessors of the reference standard	14
Analysis	**14**	Methods for estimating or comparing measures of diagnostic accuracy	13-14
	**15**	How indeterminate index test or reference standard results were handled	13-14
	**16**	How missing data on the index test and reference standard were handled	Na
	**17**	Any analyses of variability in diagnostic accuracy, distinguishing pre-specified from exploratory	11, 14
	**18**	Intended sample size and how it was determined	11, 14
**RESULTS**			
Participants	**19**	Flow of participants, using a diagram	na
	**20**	Baseline demographic and clinical characteristics of participants	eTable 1-2
	**21a**	Distribution of severity of disease in those with the target condition	eTable 1
	**21b**	Distribution of alternative diagnoses in those without the target condition	eTable 1
	**22**	Time interval and any clinical interventions between index test and reference standard	13
Test results	**23**	Cross tabulation of the index test results (or their distribution) by the results of the reference standard	4-5, Table 1,2
	**24**	Estimates of diagnostic accuracy and their precision (such as 95% confidence intervals)	4-5, Table 2
	**25**	Any adverse events from performing the index test or the reference standard	na
**DISCUSSION**			
	**26**	Study limitations, including sources of potential bias, statistical uncertainty, and generalisability	9-10

	**27**	Implications for practice, including the intended use and clinical role of the index test	8-9
**OTHER INFORMATION**			
	**28**	Registration number and name of registry	10
	**29**	Where the full study protocol can be accessed	10, Supplement
	**30**	Sources of funding and other support; role of funders	16

References

1 Montgomery SA, Åsberg M. A New Depression Scale Designed to be Sensitive to Change. *Br J Psychiatry* 1979; **134**: 382–9.

2 Kleim B, Graham B, Fihosy S, Stott R, Ehlers A. Reduced Specificity in Episodic Future Thinking in Posttraumatic Stress Disorder. *Clin Psychol Sci* 2014; **2**: 165–73.

3 Kleim B, Graham B, Bryant RA, Ehlers A. Capturing intrusive re-experiencing in trauma survivors' daily lives using ecological momentary assessment. *J Abnorm Psychol* 2013; **122**: 998–1009.
